# YBX1 mediates translation of oncogenic transcripts to control cell competition in AML

**DOI:** 10.1038/s41375-021-01393-0

**Published:** 2021-08-31

**Authors:** Florian Perner, Tina M. Schnoeder, Yijun Xiong, Ashok Kumar Jayavelu, Nomusa Mashamba, Nuria Tubio Santamaria, Nicolas Huber, Kristina Todorova, Charles Hatton, Birgit Perner, Theresa Eifert, Ciara Murphy, Maximilian Hartmann, Jessica I. Hoell, Nicolas Schröder, Sabine Brandt, Andreas Hochhaus, Peter R. Mertens, Matthias Mann, Scott A. Armstrong, Anna Mandinova, Florian H. Heidel

**Affiliations:** 1 Department of Pediatric Oncology, Dana Farber Cancer Institute, Harvard Medical School, Boston, MA USA; 2 Innere Medizin 2, Hämatologie und Onkologie, Universitätsklinikum Jena, Jena, Germany; 3 Innere Medizin C, Universitätsmedizin Greifswald, Greifswald, Germany; 4 Max-Planck-Institute for Biochemistry, Munich, Germany; 5 Department of Pediatric Oncology, Hematology and Immunology, University of Heidelberg, Heidelberg, Germany; 6 Clinical Cooperation Unit Pediatric Leukemia, DKFZ, Heidelberg, Germany; 7 Department of Hematology and Oncology, Otto-von-Guericke Medical Center Magdeburg, Magdeburg, Germany; 8 Cutaneous Biology Research Center, Massachusetts General Hospital, Harvard Medical School, Boston, MA USA; 9 Molecular Genetics Laboratory, Leibniz Institute on Aging–Fritz Lipmann Institute (FLI), Jena, Germany; 10 Core Facility, Imaging, Leibniz Institute on Aging-Fritz Lipmann Institute (FLI), Jena, Germany; 11 Department of Pediatrics 1, Martin Luther University Halle-Wittenberg, Halle, Germany; 12 Gemeinschaftspraxis für Pathologie, Schlosspark-Klinik, Berlin, Germany; 13 Clinic of Nephrology and Hypertension, Diabetes and Endocrinology, Otto-von-Guericke University, Magdeburg, Germany; 14 Leibniz Institute on Aging-Fritz-Lipmann Institute, Jena, Germany

**Keywords:** Acute myeloid leukaemia, Cancer stem cells

## Abstract

Persistence of malignant clones is a major determinant of adverse outcome in patients with hematologic malignancies. Despite the fact that the majority of patients with acute myeloid leukemia (AML) achieve complete remission after chemotherapy, a large proportion of them relapse as a result of residual malignant cells. These persistent clones have a competitive advantage and can re-establish disease. Therefore, targeting strategies that specifically diminish cell competition of malignant cells while leaving normal cells unaffected are clearly warranted. Recently, our group identified YBX1 as a mediator of disease persistence in *JAK2*-mutated myeloproliferative neoplasms. The role of YBX1 in AML, however, remained so far elusive. Here, inactivation of *YBX1* confirms its role as an essential driver of leukemia development and maintenance. We identify its ability to amplify the translation of oncogenic transcripts, including MYC, by recruitment to polysomal chains. Genetic inactivation of *YBX1* disrupts this regulatory circuit and displaces oncogenic drivers from polysomes, with subsequent depletion of protein levels. As a consequence, leukemia cells show reduced proliferation and are out-competed in vitro and in vivo, while normal cells remain largely unaffected. Collectively, these data establish YBX1 as a specific dependency and therapeutic target in AML that is essential for oncogenic protein expression.

## Introduction

Cold-shock proteins (CSPs) are a family of multifunctional DNA/RNA binding proteins that contain a highly conserved nucleic acid binding domain called the cold shock domain. YBX1 is a pleiotropic DNA and RNA binding protein that modulates translation, RNA-stability, mRNA splicing, transcription or cell signaling depending on cell type and genetic background [1–10]. In humans, eight members of the CSP-family are described: YBX1, YBX2, YBX3, CARHSP1, CSDC2, CSDE1, LIN28A and LIN28B [6]. Several of the mammalian CSP-family members promote malignant transformation or cancer progression [1–3, 11] and impact diverse inflammatory processes [6, 7]. Initially, the CSP family had been identified in bacteria as proteins required for stress responses. Upon rapid temperature decline CSPs facilitate resistance to translational stress as a consequence of changes in mRNA secondary structures [12–14]. One of the most highlighted functions of YBX1 is its ability to adapt malignant cells to hypoxic stress [1, 2, 15]. YBX1 binds and stabilizes oncogenic RNAs in the context of hypoxia [2] and directly mediates translation of HIF1a transcripts [1, 15]. Recently, our group reported on a novel role of YBX1 in JAK2-mutated myeloproliferative neoplasms (MPN) [16]. During JAK-inhibitor treatment, YBX1 safeguarded splicing of transcripts essential for signal transduction. Genetic inactivation of *YBX1* led to a significant increase in mis-splicing of MAPK/ERK pathway members and to eradication of otherwise persistent MPN cells [16]. Of note, YBX1 was not primarily required for proliferation or survival of *JAK2*-mutated cells.

So far, the functional role of cold-shock proteins in AML had not been investigated in detail. Here, we aim to assess the functional relevance and mechanistic role of cold shock proteins, and specifically YBX1, in acute myeloid leukemia (AML) in vitro and in vivo.

## Materials and methods

### Animal models

Mice were housed under pathogen-free conditions in the Animal Research Facility OvGU, Magdeburg and University Hospital Jena, Germany. All experiments were conducted after approval by the Landesverwaltungsamt Sachsen-Anhalt (42502-2-1279 UniMD) and Thüringen (02-030/2016). Generation of conventional [17] and conditional [16] mouse models for genetic inactivation of *Ybx1* has been described before. Retroviral induction of leukemia was performed as published previously [18, 19]. The experimental details for the experiments conducted in murine leukemias and xenograft systems are outlined in detail in the supplementary methods section.

### RNA sequencing

RNA was isolated from cultured cells using the Qiagen RNeasy Mini kit or from polysomal fractions using TRIZOL as previously described [4]. Subsequently, mRNAs were purified using the “NEBNext^®^ Poly(A) mRNA Magnetic Isolation Module” followed by RNAseq library preparation using the “NEBNext^®^ Ultra™ RNA Library Prep Kit for Illumina^®^” according to the manufacturer’s instruction. Sequencing was performed at Dana-Farber Cancer Institute (NexSeq, 37 bp, paired end) or at Genewiz (HiSeq, 150 bp, paired end) (Illumina, South Plainfield, NJ, USA).

### Mass spectrometry

MOLM13 cell pellets from growing cultures were washed in PBS and lysed as previously described [20] before trypsin digest. A nanoflow HPLC (EASY-nLC1000, Thermo Fisher Scientific) coupled to an Orbitrap Exploris 480 Mass Spectrometer (Thermo Fischer Scientific) via a nans electrospray ion source was utilized for the sample analysis. Peptide calling and quantification was performed as previously established [21–23]. A detailed description of the procedure is provided in the Supplementary Methods section.

### CRISPR-Cas9 screening

Paired human genome-scale CRISPR-Cas9 screening libraries (H1/H2) were a gift from Dr. Xiaole Shirley Liu (Addgene #1000000132). The H1 and H2 libraries cover protein coding genes of the genome with a total of 10 guide RNAs per gene. Lentivirus was produced using each separate library pool and used to transduce each 4 × 10^8^ MOLM13 cells harboring a knockout of *YBX1* (YBX1-sgRNA1, pLKO5.GFP) or non-targeting control at low MOI. 48 h after library transduction cells were selected with puromycin. After 3 d of puromycin selection a baseline sample was collected, and cells were cultured in duplicates for 12 d (splitting and counting every 3 d) before harvest of the terminal samples. Subsequently, genomic DNA was isolated using phenol-chloroform extraction. Guide-RNA amplicon libraries were prepared and data analysis using MAGeCK MLE was performed as previously described [24–26].

## Results

### YBX1 is a pan-cancer dependency and drives cell proliferation in human and murine models of AML

Given the fact that RNA-binding proteins may exert different functions depending on the cellular context, we employed functional and descriptive screening methods to investigate mechanisms by which CSPs may influence cellular homeostasis in AML.

**Fig. 1:**
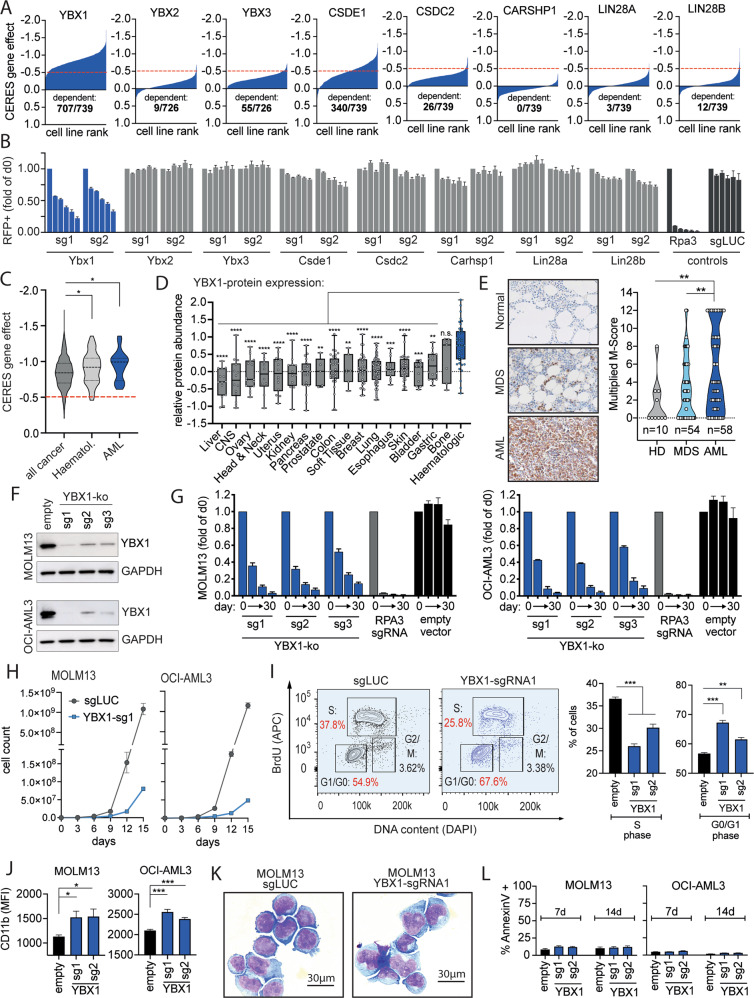
YBX1 is a pan-cancer dependency and drives cell proliferation in AML. **A** Waterfall plots depicting the gene-dependency of each cold-shock protein coding gene in cell lines from the cancer dependency map portal (Achilles program, depmap.org). Cell lines are sorted by dependency rank (increasing dependency along the *x* axis), the CERES gene-effect score is shown on the *Y* axis. The red dotted lines display the arbitrary CERES score of −0.5, which is generally considered as a cutoff for a relevant gene-dependency. **B** Bar graphs displaying the data from a CRISPR-Cas9 cell competition assay showing the effect of deletion of each cold-shock protein family member over time. Members of the CSP-family were knocked out using single guide RNAs in murine MLL-AF9 transformed AML cells and the chimerism of knockout and wild-type cells over time is visualized in the graphs. The bars within a block of each guide RNA represent the chimerism at day 0, 3, 6, 9, 12 and 15. A decrease in the % of RFP + cells (shown on the *Y* axis) over time, as seen for YBX1, reflects a competitive disadvantage of cells that harbor the respective knockout. **C** Violin plots showing the gene-dependency of YBX1 (Achilles program, depmap.org) in all cancer types compared to all hematologic cancers and AML. Statistical analysis via unpaired *t* test, *p < 0.05. **D** Box plots showing YBX1 relative protein expression among cell lines from the cancer cell line encyclopedia (Mass-spec proteome analysis, data derived from depmap.org). Statistical analysis via unpaired *t* test, **p < 0.01, ***p < 0.001, ***p < 0.0001. **E** Immunohistochemistry for YBX1 in bone marrow of healthy donors (HD), patients with myelodysplastic syndrome (MDS) and patients with AML. Left side: representative pictures of the analyzed bone marrow histology sections (brown color: anti-YBX1). Right side: Violin-plot sowing the respective pathological scoring (Multiplied M-scores) among all specimens analyzed. Statistical analysis was performed using Mann–Whitney *U* Test, **p < 0.01. **F** Western-blot showing YBX1-protein levels in MOLM13 and OCI-AML3 cells after knockout using 3 different sgRNAs compared to empty vector control. **G** Bar graphs showing data from a CRISPR-Cas9 cell competition assay in MOLM13 and OCI-AML3 cells after YBX1 deletion using 3 different sgRNAs compared to knockout of RPA3 (positive control) or empty vector (negative control) at 0, 10, 20 and 30 days after starting the competition assay. **H** Growth curves of MOLM13 and OCI-AML3 cells after deleting YBX1 using sgRNA1 compared to non-targeting control (sgLUC) over the course of 15 days. **I** Flow-cytometry-based cell-cycle analysis in MOLM-13 cells after genetic inactivation of *YBX1* using the BrdU assay. Left panel: representative FACS plots. Right panel: bar graphs showing quantitative analysis of cells detected in S- and G0/G1 phase. Unpaired *t* test, **p < 0.01, ***p < 0.001. **J** Flow cytometry-based assessment of CD11b surface expression of MOLM-13 and OCI-AML3 cells after knockout of *YBX1* using 2 different sgRNAs. Unpaired *t* test, *p < 0.05, ***p < 0.001. **K** Representative cytological pictures showing cell morphology of MOLM-13 cells after YBX1 knockout compared to control (empty; Quick-Dip staining kit, JORVET, Loveland, CO, USA). **L** Apoptosis assay using Annexin V (Biolegend, San Diego, CA, USA) in MOLM-13 and OCI-AML3 cells after knockout of *YBX1* using 2 different sgRNAs (day 7 and 14 after YBX1 knockout).

To generate insights into functional properties of different CSPs on a pan-cancer scale we utilized publicly available functional genomic datasets. Gene-dependency data from genome-wide CRISPR-Cas9 screens in over 700 cancer cell lines [27] indicated a pan-cancer dependency only for YBX1 (Fig. 1A). Of note, AML cell lines were particularly sensitive to its inactivation (Fig. 1C). In order to validate these observations, we defined CSP-specific dependencies in AML cells using an arrayed CRISPR-Cas9 based negative selection screen (Supplementary Fig. 1A). Consistent with the public pan-cancer screening data, murine MLL-AF9 transformed AML cells [28] showed a relevant gene-dependency only on Ybx1 (Fig. 1B). Analysis of a recently published large-scale proteome dataset covering 375 cell lines of the Cancer Cell Line Encyclopedia [29] for CSP-family expression showed that YBX1 and CARHSP1 are specifically overexpressed in hematologic malignancies (Fig. 1D; Supplementary Fig. 1B). Similarly, gene-expression of YBX1, CARHSP1 and YBX2 was shown to be elevated in a set of primary AML patient samples [30] (Supplementary Fig. 1C). Since YBX1 was particularly upregulated and functionally relevant we aimed to validate our findings by immunohistochemistry in bone marrow (BM) biopsies from patients. Compared to healthy donors (HD), patients with myelodysplastic syndrome (MDS) or AML showed increased expression during disease progression with the highest scores documented in the AML specimens (Fig. 1E). We further validated these findings in two different human AML cell lines (MOLM13, OCI-AML3) using 3 sgRNAs targeting YBX1 that potently reduced protein expression (Fig. 1F). *YBX1*-inactivation led to gradual out-competition of guide infected cells (Fig. 1G). Loss of cell competition could be attributed to impaired proliferative capacity and delayed S-phase entry of YBX1-deficient AML cells (Fig. 1H, I). Furthermore, AML cell lines showed discrete immunophenotypic and morphological signs of differentiation (Fig. 1J, K) while induction of apoptosis was not observed (Fig. 1L). To validate our findings, we used RNAi to genetically inactivate *YBX1* in a larger panel of AML cell lines. For both YBX1 shRNAs 6/8 AML cell lines showed >70% reduction in cell proliferation (Supplementary Fig. 2A) but no consistent increase in apoptosis. Signs of myeloid differentiation could also be detected but appeared rather inconsistent and not clearly associated with cellular responses (Supplementary Fig. 2B).

### YBX1 is essential for development and maintenance of AML in vivo

**Fig. 2:**
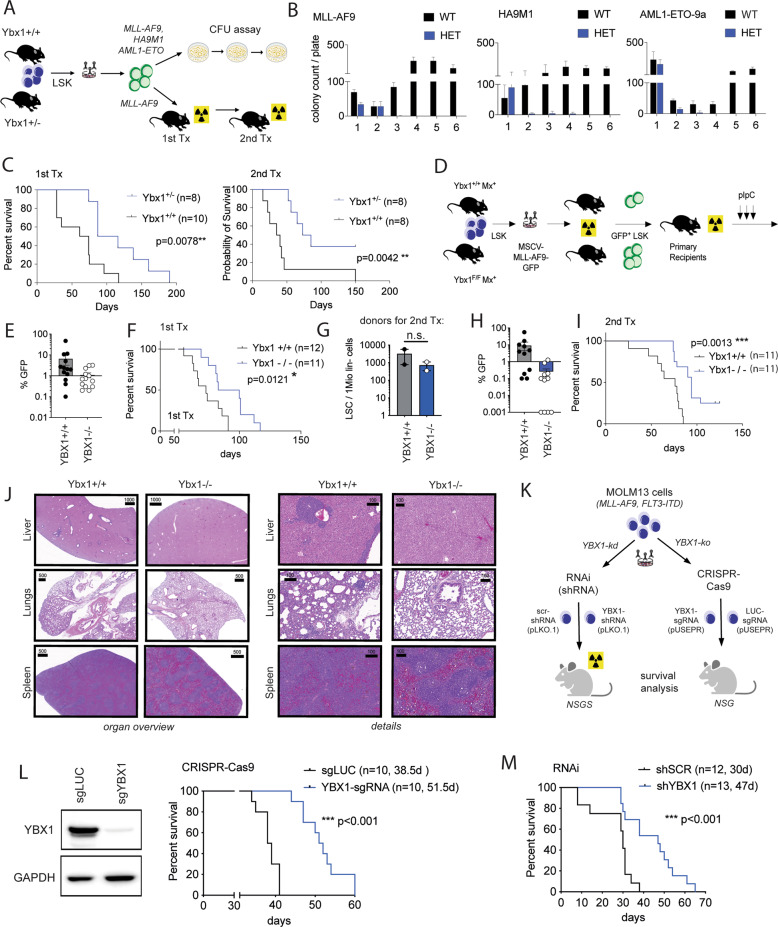
YBX1 is essential for development and maintenance of AML in vivo*.* **A** Flow-scheme depicting the experimental procedures performed using the Ybx1 straight knockout mouse model for assessment of Ybx1 function in development of AML. **B** Bar graphs showing the number of AML colonies in a methylcellulose-based colony-formation assay in Ybx1 + /+ and Ybx1 + /− AML cells after retrovirally mediated leukemic transformation with MLL-AF9, HOXA9-MEIS1a (HA9M1) or AML1-ETO9a. The numbers on the *X* axis correspond to the rounds of plating. **C** Survival-curves of primary (left) and secondary (right) recipient animals after transplantation of MLL-AF9 transformed AML cells harboring a heterozygous deletion of Ybx1 (YBX1 + /−) or WT (Ybx1 + /+). **D** Scheme depicting the experimental procedures for assessment of Ybx1 function in maintenance of AML. **E** Bar graphs showing the % of GFP + cells (MLL-AF9 expressing leukemia cells) in the peripheral blood of Ybx1 + /+ and Ybx1 + /− primary recipient animals 4 weeks after transplantation. **F** Survival of primary recipient animals transplanted with Ybx1 + /+ and Ybx1 + /− MLL-AF9 driven AML. **G** Bar graphs showing the LSC frequency (number of GFP + Kit + cells per 1Mio lin- viable bone marrow cells) in primary recipient animals that were used as donors for secondary recipients. **H** Bar graphs showing the % of GFP + cells (MLL-AF9 expressing leukemia cells) in the peripheral blood of Ybx1 + /+ and Ybx1 + /− secondary recipient animals 2 weeks after transplantation. **I** Survival of secondary recipient animals transplanted with Ybx1 + /+ and Ybx1 + /− MLL-AF9 driven AML. **J** Histological pictures (H&E staining) of liver, lung and spleen of representative secondary recipient animals that were transplanted with a Ybx1 + /+ and Ybx1 + /− AML. On the left side is the organ overview, the right side shows the same sections in higher magnification for the visualization of microscopic structures. The numbers on the scale bars show the respective scale in µm. **K** Scheme depicting the experimental procedures for CRISPR-Cas9-mediated knockout and RNAi mediated knockdown of *YBX1* in human MOLM-13 cells before transplantation into xenograft mice. **L** Left side: Western blot showing knockout efficiency before transplantation. Right side: survival curve for xenograft mice injected with MOLM13 cells after CRISPR-Cas9 mediated knockout of YBX1. **M** Survival curve of mice transplanted with MOLM13 after RNAi mediated knockdown of YBX1 (shYBX1) or non-targeting control.

Reduction of YBX1 expression by RNAi in primary MLL-AF9 (MA9) transformed murine leukemic cells resulted in decreased colony formation capacity and a significant delay of disease development in vivo (*p* = 0.0446 *; Supplementary Fig. 3A, B). In order to determine the relevance of YBX1 for AML development in a more sophisticated genetic system, we used a retroviral model of leukemic transformation in a conventional YBX1 knockout mouse model [17] in which exon 3 is genetically deleted leading to loss of a functional protein. As homozygous deletion of Ybx1 is embryonically lethal, we compared heterozygous animals to wildtype controls. Bone marrow (BM) cells of the respective donor animals were isolated as published before [19, 31] and Ybx1 + /+ or Ybx1 + /− Lin^−^Kit^+^Sca1^+^ (LSK) cells were transduced with MLL-AF9 (MA9), HoxA9-Meis1a or AML1-ETO. Transformed cells were investigated by serial re-plating in methylcellulose to assess colony formation and self-renewal capacity in vitro (Fig. 2A). As expected, Ybx1 + /+ cells showed increased self-renewal. In contrast, Ybx1 + /− cells failed to sustain colony growth beyond 3 rounds of serial re-plating for all oncogenes investigated (Fig. 2B). To investigate whether Ybx1 is required for leukemia development in vivo, Ybx1 + /+ and Ybx1 + /− LSK cells were transduced with the MA9 fusion oncogene and a total of 7 × 10^4^ GFP + cells were injected into primary recipient hosts (Fig. 2A). Recipients of Ybx1 + /− cells showed delayed disease onset and significantly prolonged survival (median survival of MA9-Ybx1 + /+ 67 days; MA9-Ybx1 + /− 101 days; *p* = 0.0078**) (Fig. 2C, left panel). Likewise, secondary recipients of Ybx1 + /− cells showed prolonged survival (median survival of MA9-Ybx1 + /+ 37 days; MA9-Ybx1 + /− 90 days; *p* = 0.0042**) and 3/8 (37.5%) of animals failed to establish leukemia within 150 days (Fig. 2C, right panel). To assess for a potential therapeutic index and for the role of Ybx1 in normal HSPC function, Ybx1 + /− and Ybx1 + /+ cells were transplanted into primary recipient hosts in a competitive manner. We found no loss of function in heterozygous Ybx1 cells when competing against wildtype controls as indicated by stable peripheral blood (PB) chimerism over 16 weeks in primary and secondary recipient hosts (Supplementary Fig. 3C). Furthermore, the composition of hematopoietic stem- and progenitor cells (HSPCs) in the BM of Ybx1 + /− mice was not altered compared to Ybx1 + /+ animals (Supplementary Fig. 3D). These findings indicate that heterozygous deletion of Ybx1 impairs leukemia development in vivo while it does not affect normal HSPC function to a major extent. To confirm the role of Ybx1 in leukemia maintenance, we used a conditional knockout mouse model that was recently published by our group [16] and allows for conditional deletion of Ybx1 after leukemia onset. Here, exon 3 of Ybx1, that encodes for a part of the conserved cold shock domain was genetically deleted through activation of Mx1-Cre-recombinase (Fig. 2D). Inactivation of Ybx1 by pIpC injections after engraftment of leukemic cells in primary recipient mice resulted in a delay of leukemia onset (Fig. 2E, Supplementary Fig. 3E) and prolongation of survival (median survival of MA9-Ybx1 + /+ 73 days; MA9-Ybx1−/− 91.5 days; *p* = 0.0121*) (Fig. 2F). Of note, the frequency of leukemic stem cells was not significantly decreased in the primary recipient hosts transplanted with Ybx1−/− leukemia cells (Fig. 2G) compared to WT controls. This finding indicates a competitive disadvantage rather than exhaustion of AML-LSCs. In secondary recipient hosts, Ybx1−/− leukemias showed reduced proliferation (Fig. 2H, Supplementary Fig. 3F), failed to re-establish leukemia in 3/10 recipients (Fig. 2H, I) and significantly prolonged survival compared to Ybx1 + /+ controls (median survival of MA9-Ybx1 + /+ 76 days; MA9-Ybx1−/− 94 days; *p* = 0.0013**) (Fig. 2I). Histopathological analysis of internal organs of Ybx + /+ recipients showed expected infiltration in liver, spleen and lungs (Fig. 2J, left panel). In contrast, in Ybx1−/− mice sacrificed without clinical signs of leukemia at day 150, no relevant leukemic organ infiltration could be observed (Fig. 2J, right panel).

**Fig. 3:**
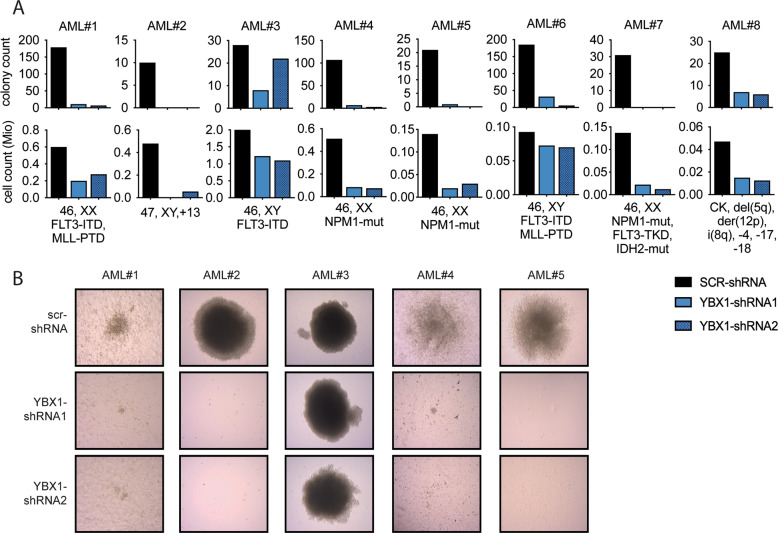
YBX1 is a dependency in primary human AML patient samples. **A** Bar graphs showing the colony counts (top panel) and cell counts (bottom panel) of primary human AML patient samples after genetic inactivation of YBX1 (YBX1-shRNA1/2) or control (SCR-shRNA). After lentiviral transduction cells were plated in methylcellulose (MethoCult™ H4034 Optimum, Stemcell Technologies, Vancouver, Canada) supplemented with 1 µg/ml puromycin at a density of 100.000 cells/4 ml of methylcellulose in a 6-well plate. Colonies were counted and analyzed at 10-14d after plating. **B** Representative pictures of colonies from 5 primary AML patients after YBX1 inactivation and plating in methylcellulose.

To validate the functional impact of YBX1 depletion in human AML in vivo, we performed a CRISPR-Cas9 mediated knockout as well as shRNA-mediated knockdown of YBX1 in MOLM13 cells and assessed leukemia dynamics after transplantation in humanized mice (Fig. 2K). Inactivation of YBX1 delayed disease progression in both models and led to a significantly improved overall survival (CRISPR: median survival of sgLUC: 38 days; sgYBX1 51 days; *p* < 0.001***; RNAi: median survival of shSCR: 30 days; shYBX1: 47 days; *p* < 0.001***) (Fig. 2L, M). To further assess the effects of *YBX1*-depletion in primary AML-specimens, we used BM aspirates from 8 AML patients reflecting a diverse spectrum of molecular- and cytogenetic aberrations. Depletion of *YBX1* led to decreased cell numbers and colony formation in vitro (Fig. 3A, B). Together, these findings confirm a functional requirement of YBX1 for the development and maintenance of murine and human AML in vitro and in vivo.

### YBX1 maintains an oncogenic protein network in AML cells at the post-transcriptional level

**Fig. 4:**
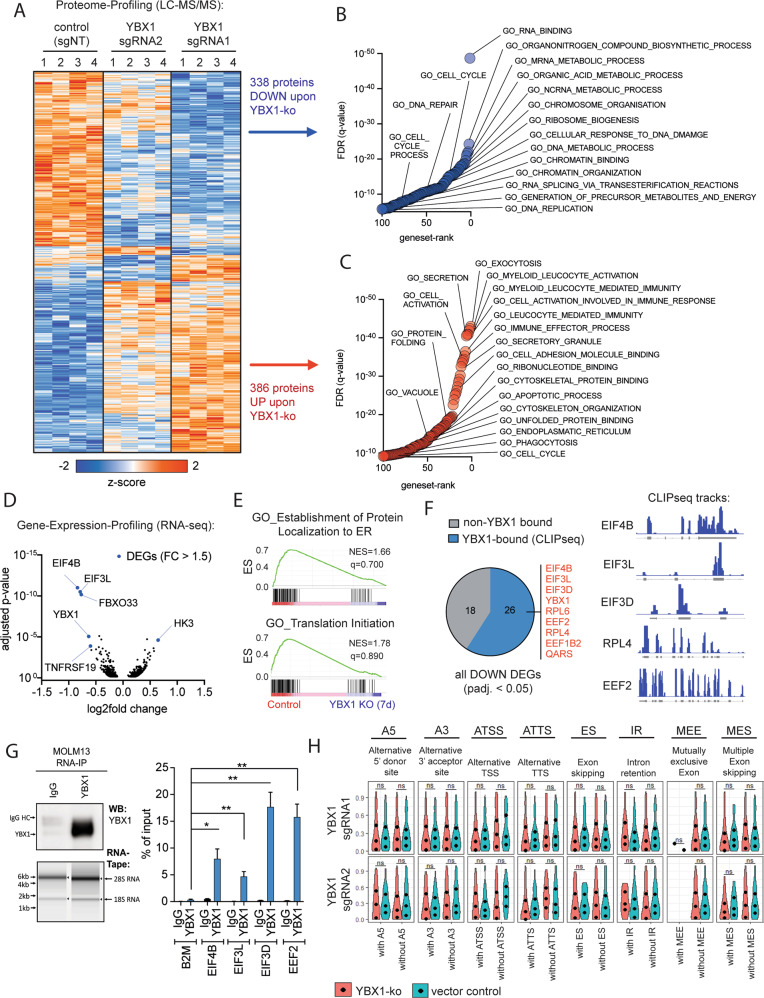
YBX1 maintains an oncogenic protein network in AML at the post-transcriptional level. **A** Heatmap derived from hierarchical clustering of proteins that were detected as being differentially abundant in mass-spectrometry based whole proteome analysis. *YBX1* was inactivated in MOLM-13 cells using CRISPR-Cas9 mediated knockout (2 different sgRNAs). Lentiviral transductions were performed in quadruplicates and 2 × 10^6^ cells per replicate were FACS-sorted 14 days after transduction for the strongest guide-expressing cells (RFP-high) before cell lysis, trypsin digest and analysis via mass-spectrometry. Proteins significantly up- or down-regulated with both guide RNAs (ANOVA *p* < 0.05) were considered. **B** Gene-set enrichment analysis for Gene-ontology terms on proteins that were significantly down-regulated in the whole proteome analysis. Selected terms are annotated. **C** Gene-set enrichment analysis for Gene-ontology terms on proteins that were significantly up-regulated in the whole proteome analysis. Selected terms are annotated. **D** Volcano-plot of differentially expressed genes in RNAseq at day 7 after knockout of *YBX1* by CRISPR-Cas9 (2 different guide RNAs). Every dot represents a gene with a *p* value below 0.05. The dots highlighted in blue are genes among those that show a fold change above 1.5. **E** Two of the top Gene-ontology terms that were found to be lost in GSEA of RNAseq data in MOLM-13 cells with knockout of *YBX1*. **F** Depiction of differentially expressed genes from RNAseq in *YBX1*-deficient MOLM-13 cells that were previously identified as RNA-binding targets of YBX1 [2]. Left: Pie chart showing the proportion of DEGs that are known binding partners of YBX1 (blue) in relation to genes that have not been shown to bind to YBX1 (gray). Right: iCLIP-seq IGV-tracks of selected DEGs, that are established binding partners of YBX1 [2]. **G** RIP-qPCR of YBX1 from MOLM-13 cells. Left: Western-blot and RNA-tape bands as a quality control of the YBX1-enrichment during immunoprecipitation and RNA integrity post IP. Right: results of RIP-qPCR shown as % of enrichment over input validating the binding of EIF4B, EIF3L, EIF3D and EEF2 to YBX1 in MOLM-13 cells. **H** Analysis of differential splicing in YBX1-deficient cells (sgRNA1/sgRNA2) compared to vector control. The computational analysis to call alternative splicing events was performed using the “IsoformSwitchAnalyzeR”-package [33].

For an unbiased assessment of protein networks that are regulated by YBX1, we performed whole proteome profiling using mass-spectrometry. Statistical analysis revealed a total of 386 significantly up- and 338 downregulated proteins (Fig. 4A). Gene-ontology analysis showed that YBX1 inactivation led to reduced abundance of proteins associated with cellular homeostasis of proliferating cells, including RNA- and DNA-metabolism, splicing, chromatin- and protein-homeostasis and cell division (Fig. 4B). Conversely, signatures associated with proteins upregulated in response to *YBX1* deletion were associated with myeloid differentiation and innate immunity, reflecting cell cycle arrest and loss of immaturity (Fig. 4C).

To determine how YBX1 is regulating the abundance of these proteins, we performed RNA-sequencing 7 days after knockout. Interestingly, the number of differentially expressed genes (DEGs, fold-change >1.5, adjusted *p* < 0.05) appeared rather small, with only 6 genes meeting the criteria for significance (Fig. 4D). When considering all genes with an adjusted *p* value below 0.05 irrespective of the fold-change, 75 genes reached statistical significance (Supplementary Fig. 4A). Gene-set-enrichment analysis (GSEA) revealed signatures associated with translation-initiation (Fig. 4E). The ability of YBX1 to impact gene expression by binding and stabilizing mRNAs has been previously demonstrated [2, 5]. Therefore, we aimed to assess if the transcripts that are regulated by YBX1 on the RNA level may be targets of YBX1-mRNA binding. Utilizing a previously published iCLIPseq dataset [2] we confirmed that the majority of transcriptionally downregulated genes are substrates of YBX1-binding (Fig. 4F). Using RNA-immunoprecipitation followed by quantitative real-time PCR (RIP-qPCR) YBX1-binding to 4 of those transcripts encoding for proteins involved in translation initiation and elongation could be validated (Fig. 4G). Our group had previously demonstrated, that YBX1 is safeguarding splicing in MPN and that deletion of YBX1 led to a global increase in miss-splicing affecting specific transcripts that are required for disease persistence [16]. Therefore, we assessed for differential splicing and miss-splicing events in our RNAseq dataset. In contrast to our previous findings in *JAK2*-mutated cells, no global alterations in alternative splicing events could be detected after *YBX1* deletion in human AML cells (Fig. 4H). Furthermore, we aimed to assess for DNA-binding of YBX1 and its postulated potential to act as a transcription factor. In order to determine localization and distribution of YBX1 over the genome, ChIP-sequencing was performed. Approximately 50% of YBX1-specific peaks were localized at regions mapping to genes, with the majority of peaks localized at intronic regions (Supplementary Fig. 4B). However, genes that were differentially expressed following *YBX1* deletion did not show relevant YBX1 binding. Notably, YBX1-DNA binding to a specific gene may be associated with a repressive function, since we detected a trend for YBX1-bound genes to show increased expression following *YBX1* deletion (Supplementary Fig. 4C).

### YBX1 mediates translation in a transcript-dependent manner

**Fig. 5:**
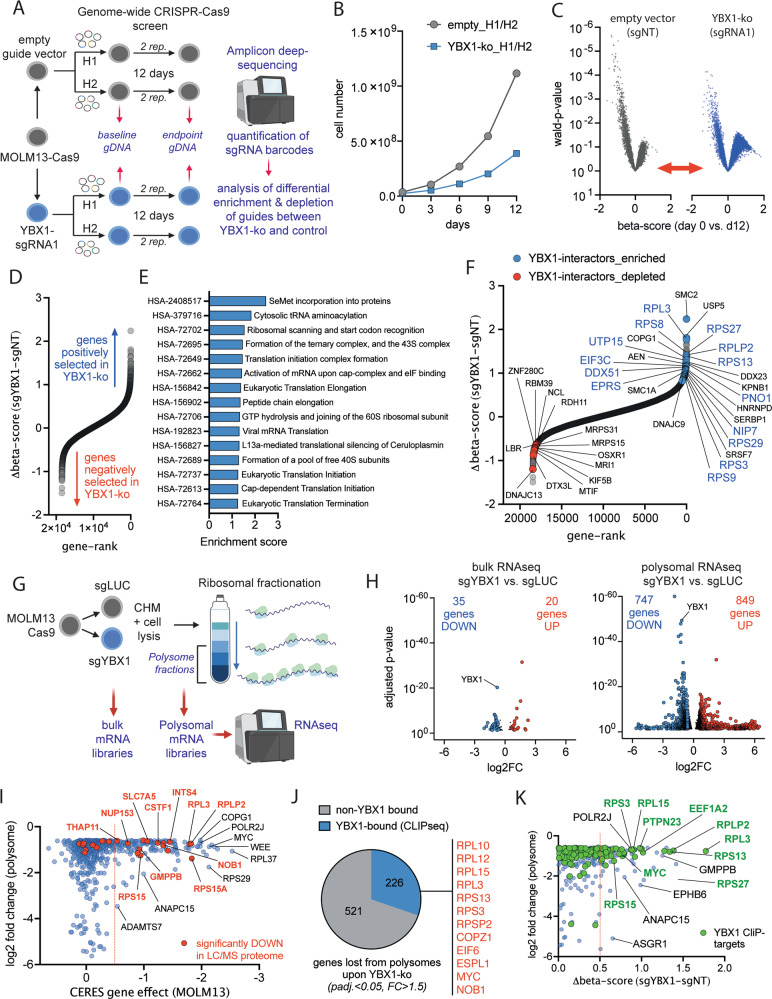
YBX1 modulates translation in a transcript-dependent manner. **A** Schematic depicting the experimental procedure to conduct the genome-wide CRISPR-Cas9 screen. MOLM13 cells were transduced YBX1-sgRNA1 or non-targeting control in the p.LKO5-GFP vector system. Cells were subsequently sorted for GFP^high^ expressing cells and expanded to facilitate genome-wide screening. Lentiviral transductions with 2 paired human whole genome-libraries (H1/H2) [25] were performed at low multiplicity of infection (MOI) and cells were selected with puromycin for 3 days. The screen was carried out for 12 days after puromycin selection was completed and the baseline DNA sample was harvested. **B** Growth curve of MOLM-13 cells with a YBX1-KO (blue line) or empty vector control (gray line) during the CRISPR-Cas9 screen for the estimation of the selective pressure applied during the screen. **C** Volcano-plots showing the distribution of genes being enriched (positive beta-scores) or depleted (negative beta-scores) in the genome-wide CRISPR-Cas9 screen in cells harboring a YBX1-KO compared to control. **D** Dot-plot of ranked differential CRISPR-Cas9 screening hits between the YBX1-KO and control condition. The *X* axis shows the gene-rank, on the *Y* axis the differential beta scores (Δbeta-scores) are plotted. **E** Top15 REACTOME-terms showing differential enrichment in the CRISPR-Cas9 screening dataset between the YBX1-KO and the control condition. **F** Dot-plot of ranked differential CRISPR-Cas9 screening hits between the YBX1-KO and control condition. Highlighted as red or blue dots are the genes among the top 500 differentially enriched or depleted genes in the YBX1-KO condition, that have previously been identified as binding partners of YBX1 in IP-Mass spec [16]. **G** Schematic depicting the experimental procedure underlying the polysomal RNA profiling in MOLM-13 harboring a YBX1-KO or non-targeting control (sgLUC). MOLM13 cells were transduced with YBX1-sgRNA (or NT-control), selected with puromycin and expanded for 14 days prior to polysomal fractionation followed by RNA-sequencing. **H** Volcano-plot of differentially expressed genes in RNAseq 14 days after knockout of *YBX1* by CRISPR-Cas9 (YBX1-sgRNA1). Left: DEGs in YBX1-KO cells compared to control when sequencing the total cellular mRNA content. Right: DEGs in YBX1-KO cells when sequencing mRNAs that are bound to polysomal chains. **I** Correlation between the magnitude of gene-loss from the polysomal fractions (*Y* axis: log2FC) upon YBX1-KO and the dependency of the respective genes from the cancer dependency map portal (*X* axis: CERES gene effect, depmap.org). The red dotted line marks the arbitrary cutoff of genes that are generally considered functional dependencies. Genes that are labeled in red are genes that have been identified as differentially expressed hits in proteome screening. **J** Pie chart showing the proportion of genes that were found to be lost from polysomes and are known binding partners of YBX1 (blue) in relation to genes that have not been shown to bind to YBX1 (gray). **K** Correlation between the magnitude of gene-loss from the polysomal fractions (*Y* axis: log2FC) upon YBX1-KO and the differential dependency of the respective genes in our CRISPR-Cas9 screen in YBX1-KO vs. control cells. Genes that are labeled in green are genes that have been identified as RNA-targets of YBX1 in iCLIPseq [2].

In order to generate a global view on the functional properties of YBX1 in AML, we performed a genome-wide CRISPR-Cas9 screen in MOLM13 cells comparing the genetic vulnerabilities of YBX1-knockout and control cells (Fig. 5A). This functional genomics approach enabled us to screen in an unbiased manner for cellular networks that are specifically affected by YBX1 loss. As expected, genetic deletion of *YBX1* reduced cellular proliferation thus providing the required selective pressure to conduct the screen (Fig. 5B). Following Next-Generation Sequencing, alignment and quantification of each guide-RNA barcode to the respective guide library, *p* values and corresponding beta-scores were calculated for each gene (Fig. 5C). Positive beta scores represent an enrichment of guides targeting a certain gene over time, typically being interpreted as a tumor-suppressor-like function, while negative beta-scores represent selective dependencies resulting in out-competition. The beta score of each gene in the non-targeting (NT) control condition was then subtracted from the respective score in the *YBX1*-knockout condition to generate a Δbeta-score that reflects differential dependency (Fig. 5D). When performing GSEA for REACTOME-terms on the ranked list of Δbeta-scores, the top 15 enriched terms reflected pathways and functions associated with translational initiation and elongation (Fig. 5E). Most genes associated with these terms represent functional dependencies in the NT-control condition, since translation mediators and ribosomal subunits are important housekeeping genes but lose this specific gene-dependency in the YBX1-knockout setting (Supplementary Fig. 5A). This finding suggests that YBX1 exerts its function via these molecules. Of note, among the top differential dependencies, several targets had previously been identified as protein binding partners of YBX1 [16], highlighting the power of functional genomic screening for the identification of functional molecular networks (Fig. 5F).

**Fig. 6:**
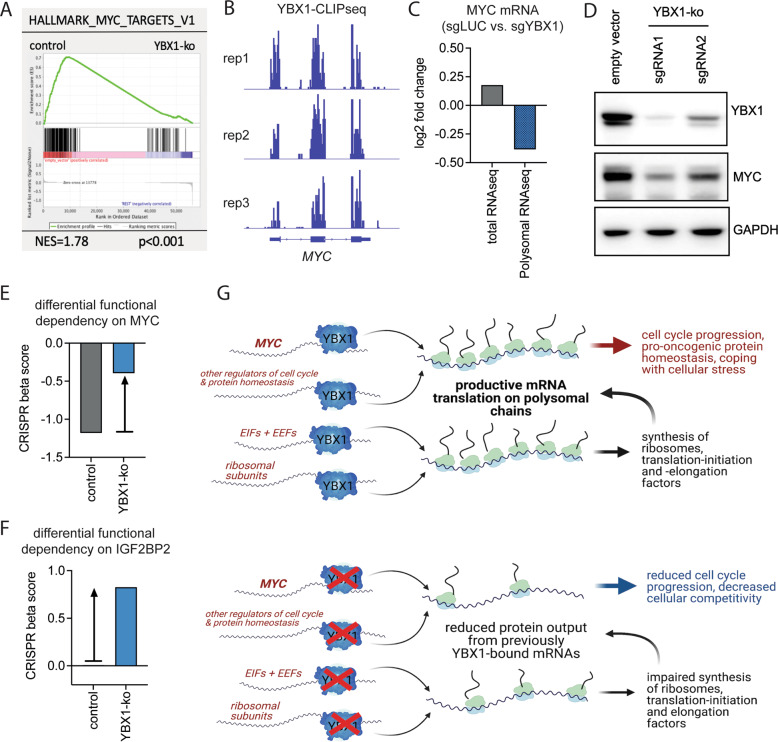
YBX1 regulates MYC by modulating polysomal recruitment. **A** Geneset enrichment analysis showing a loss of a MYC target gene signature in MOLM-13 cells harboring a knockout of *YBX1* (sgRNA1/2, day 7 after lentiviral transduction). **B** Representative iCLIPseq tracks over the MYC gene from 3 independent replicates, showing binding of YBX1 to MYC in breast cancer [2]. **C** Bar graph depicting gene expression changes of MYC in total RNAseq (gray) and polysomal RNAseq, showing that a reduction in MYC-expression appears to be restricted to the polysomal fractions rather than to the unfractionated total RNA. **D** Western Blot showing the protein expression of MYC in relation to the knockout efficiency of YBX1 using 2 different sgRNAs in MOLM-13-Cas9 cells. **E** Bar graph visualizing the beta scores of MYC in control MOLM-13 cells compared to YBX1-KO cells. **F** Bar graph visualizing the beta scores of IGF2BP2 in control MOLM-13 cells compared to YBX1-KO cells. **G** Schematic showing the proposed model of YBX1-action in AML by binding to its target RNAs and mediating their recruitment to polysomal chains to drive productive translational output.

In order to assess for the ability of YBX1 to influence translation of mRNAs, we performed transcriptomic profiling from purified ribosomal fractions (Fig. 5G, Supplementary Fig. 5G, 6). Recruitment of mRNAs to polysomal chains is a major mechanism to increase the output of protein synthesis per mRNA molecule and is therefore considered a crucial determinant of translation efficiency. Consistent with our observations from RNA-sequencing (day 7), the number of DEGs in the bulk RNAseq-sample appeared rather limited (Fig. 5H, left panel). Genes showing reduced expression were predominantly translation initiation factors with EIF4B showing the strongest reduction on the protein level (Supplementary Fig. 5B–D). In contrast, we observed a large number of genes being differentially expressed within the polysomal fractions (Fig. 5H, right panel). The number of DEGs detected after polysomal fractionation was about 20-fold increased, compared to bulk mRNA and some genes showed a high magnitude of change. Of note, forced expression of EIF4B as the single initiation factor that was consistently and strongly affected by YBX1-ko on the total RNA and protein level was not sufficient to rescue the competitive disadvantage of YBX1-inactivation (Supplementary Figure 5E,F), suggesting a direct impact of YBX1 on polysomal transcript recruitment. Relevant YBX1-targets on polysomes were validated on the protein level by Western blot (Supplementary Fig. 5H). To identify candidates that are lost from polysomes and represent relevant functional dependencies, we integrated the magnitude of loss from the polysomes of each significantly down-regulated gene (adjusted *p* < 0.05, fold change >1.5) with the CERES gene effect score from genome-wide CRISPR-Cas9 screens (Broad-Institute, Achilles-portal). 153/747 (20.5%) of genes lost from the polysomes were shown to be functional dependencies identified by CRISPR-Cas9 editing (CERES-score < −0.5) (Fig. 5I). Importantly, a number of those genes, including cell cycle mediators and ribosome subunits showed decreased expression in global proteome analysis (Fig. 5I, highlighted in red). Furthermore, 30% (*n* = 226) of genes that were lost from the polysomes represent RNA-binding targets of YBX1 in iCLIP-sequencing analyses (Fig. 5J) [2]. Finally, we aimed to understand how genes that are lost from polysomes are associated with YBX1-dependent functional pathways. Therefore, we integrated the magnitude of loss from polysomes with the respective functional dependencies (Δbeta-scores; Fig. 5K). Here, relevant targets could be identified that were differentially recruited to polysomes and also enriched following CRISPR-Cas9 editing. Several of these targets, including MYC, were also CLIP-targets of YBX1. GSEA showed significant loss of the MYC target gene signature (Fig. 6A). Using iCLIP-sequencing it had been demonstrated, that YBX1 is consistently bound to MYC-transcripts, establishing MYC as a high confidence mRNA-binding partner of YBX1 (Fig. 6B). Importantly, genetic inactivation of *YBX1* did not affect MYC-transcript abundance in bulk RNA-sequencing (Fig. 6C). In contrast, MYC mRNA was significantly lost from the polysomal mRNA fraction upon YBX1-deletion demonstrating an involvement of YBX1 in the recruitment of MYC transcripts to polysome chains (Fig. 6C). Consequently, using two different sgRNAs that reduce YBX1 expression to a different extent, gene-dose dependent reduction in MYC expression could be confirmed (Fig. 6D). Likewise, MYC was a prominent dependency in MOLM13 cells, an effect that was significantly reduced following genetic deletion of YBX1 (Fig. 6E). The fact that MYC was identified as a relevant driver of YBX1 dependent gene expression and YBX1 is binding to MYC mRNA, indicates its role as a direct downstream effector. In line with a recent report demonstrating IGF2BP-family proteins as being critical for YBX1-binding to its target mRNAs [5], IGF2BP2-knockout was shown to mediate resistance to *YBX1*-inactivation (Fig. 6F).

Taken together, we propose, that YBX1 associates with target mRNAs, including MYC, and thereby modulates translational output by recruitment of relevant mRNAs to polysomal chains (Fig. 6G). Protein expression of MYC (among other mediators of cell cycle progression and cellular homeostasis) appears to be stabilized through YBX1 due to its preferential recruitment to polysomes. Furthermore, YBX1 may indirectly influence translation by regulating the availability of ribosomal building blocks and translation mediators (Fig. 6G). Therefore, genetic inactivation of *YBX1* impacts the translational output of transcripts on the protein level and thereby selectively modulates protein abundance of oncogenic drivers and influences proliferative capacity and cell competition in AML.

## Discussion

Identification of therapeutic targets that are tractable vulnerabilities and selective dependencies in cancer while being dispensable for normal tissues represent the ideal prerequisite for the development of cancer therapies. Cold shock protein YBX1 has been identified as a pan-cancer dependency in publicly available CRISPR-Cas9-screens and several studies in different tumor entities [1–5, 15]. Conversely, genetic inactivation of *YBX1* had no deleterious effects on normal hematopoiesis [16], making it a potentially interesting therapeutic target for cancer therapy. Consistent with recent reports [5], we have shown, that YBX1 is required for development and maintenance of human and murine AML in vitro and in vivo. Even though the cold shock domain as a common structural component of the CSPs is conserved among the family members, only YBX1 showed a potent phenotype in leukemia as well as in other cancers.

Mechanistically, we demonstrate that deletion of *YBX1* in AML shows minor impact on mRNA abundance, while having significant effects on the cellular proteome. Moreover, no relevant increase in mis-spliced isoforms could be found in AML cells after deleting YBX1, clearly distinguishing the apparent mechanisms in AML from our previous findings in MPN, where YBX1 was acting as a relevant splicing factor [16]. Using an unbiased multi-omics screening approach, we found that YBX1 mediates translation of specific transcripts in AML, which is in line with previous reports [1, 4, 15]. To the best of our knowledge this is the first report describing a global CSP regulatory network using functional genomics. Taken together, our data provide strong evidence for YBX1 acting as a cancer-specific modulator of translation in AML, while leaving total mRNA levels largely unaffected.

A recent report published by Feng and colleagues [5] complements our findings by providing novel insights into how YBX1 binds its target mRNAs in leukemia cells. YBX1 appears to bind to methylated (m^6^A) transcripts via IGF2BP-family of proteins to facilitate RNA binding and stabilization. In line with this claim, we find that deletion of IGF2BP2 confers resistance to *YBX1*-inactivation in our CRISPR-Cas9 screen. Structurally, the cold-shock domain seems to be required for both IGF2BP- and mRNA-binding of YBX1. Consistent with our findings, Feng et al. report an impact of YBX1 on MYC expression and show that its expression can rescue the phenotype evoked by inactivation of *YBX1*.

In contrast to our findings the authors assume that regulation of RNA stability represents a major mechanism of YBX1-action in AML, similar to findings described in breast cancer [2]. This assessment is based on experimental data showing that shRNA-mediated knockdown of *YBX1* can affect RNA abundance [5]. However, when we conducted parallel RNA-sequencing comparing RNAi- and CRISPR-mediated genetic inactivation of *YBX1* (to rule out a potential bias) we found regulation of RNA-stability exclusively in RNAi- but not CRISPR-treated AML cells. In RNAi-treated samples, we observed high numbers of DEGs, including MYC, BCL2 and MCL1, consistent with findings described by Feng and colleagues (Supplementary Fig. 5I). Absence of these findings in cells treated with CRISPR-Cas9 technology indicate that an intracellular defense and stress response when using RNAi may influence gene expression changes. Therefore, we assume that the mechanism of action and kinetics of YBX1 inactivation substantially influence experimental results.

Taken together, our data and the findings presented by Feng et al. establish YBX1 as a selective genetic vulnerability in leukemia without major restrictions towards specific genetic subtypes.

Of note, a novel small molecule, SU056, was recently reported to directly bind and inhibit YBX1 [32]. SU056 demonstrated activity in ovarian cancer models in vitro and in vivo and showed favorable biochemical and pharmacologic properties. The availability of this compound will allow direct targeting of YBX1 in pre-clinical models and may facilitate translation into early clinical trials in AML.

## Supplementary information

Supplementary Material and Methods

Supplementary data

## Data Availability

Raw and processed sequencing data have been made publicly available via the Gene-Expression-Omnibus platform (GEO) under the Accession numbers: GSE175713 (RNAseq), GSE175714 (Polysomal RNAseq), GSE175712 (ChIPseq). The proteomic dataset has been made available via ProteomeXchange under the identifier PXD026329.
