# Supplementary material for: YBX1 mediates translation of oncogenic transcripts to control cell competition in AML
Source: Leukemia. 2021 Aug 31;36(2):426–37. doi: 10.1038/s41375-021-01393-0 (PMC8807392; doi:10.1038/s41375-021-01393-0)
Supplement: Supplementary file 1 — Supplementary Material and Methods [file 41375_2021_1393_MOESM1_ESM.pdf]

**Supplementary material:**

**YBX1 mediates translation of oncogenic transcripts to control cell competition in AML.**

## Supplementary methods

### *Cell culture and lentiviral transduction:*

Human and murine cell lines were cultured in humidified incubators at 37°C and 5% $CO_2$ atmosphere. All cell lines were purchased from DSMZ, previously identified and tested for mycoplasma contamination every 8-12 weeks. Human cells were grown in RPMI (Gibco/Thermo Fischer Scientific, Waltham, USA) with 10% FBS (Sigma-Aldrich, St. Louis, USA) and 1% Penicillin/Streptomycin solution (Gibco/Thermo Fischer Scientific, Waltham, USA). Murine cells were cultured in "Myeloid media", supplemented with IL3, IL6 and SCF (20). Lentiviral transductions were performed as previously or established (16). A description of the procedure is provided in the supplementary methods section.

### *CRISPR-Cas9 cell competition assays:*

Murine or human cell lines were lentiviral transduced with Lenti-Cas9-2A-blast (Addgene: #73310) to stably express S. pyogenes Cas9. After selection with Blasticidine (5µg/ml) the cells were infected with lentiviral particles carrying the respective guide RNAs in the pUSEPR vector system as described before (1). Target cells were transduced with this RFP-expressing vector at a low to medium MOI resulting in a fraction of 10-50% RFP+ (guide expressing) cells. Cell competition assays were carried out in triplicates in 96-well plates and the fraction of RFP+ cells were measured at baseline (3d after transduction) and the indicated time points using a BD LSR Fortessa flow cytometer. Ratios between baseline and the respective time points were calculated and displayed as relative fractions.

**Lenti- and Retroviral particle production and transductions:** HEK-293T cells were cultured in DMEM (Gibco/Thermo Fischer, Waltham, USA) with 10% FBS (Sigma-Aldrich, St. Louis, USA). Cells were detached with Trypsin-EDTA (Gibco/Thermo Fischer, Waltham, USA), washed in pre-warmed medium and 2Mio cells were plated into 10cm cell culture treated dishes (Corning, NY, USA) and cultured to about 80% confluence for 24h. Lipofection was performed using FuGene (Promega, Madison, WI, USA) or XtremeGene (Sigma-Aldrich, St. Louis, USA). For the production of lentiviral particles, 30µl lipofection reagent was mixed with 1.8ml OptiMEM (Gibco/Thermo Fischer, Waltham, USA) and incubated for 5min. at room temp. The lentiviral expression vector, as well as the packaging plasmids psPAX2 (Addgene: #12260) and pMD2.G (Addgene #12259) were mixed at 3µg per plasmid and added to the lipofection mixture before incubation for 15min. at room temp. The cell culture medium on the 293T cell plates was carefully removed and replaced with 4ml pre-warmed culture medium. Subsequently, the lipofection mixture was added dropwise to the plate and the plate was incubated at 37°C and 5% $CO_2$. After 24h, the medium was removed and replaced with 10ml fresh pre-warmed medium. The viral particles were harvested after another 24h and filtered through a 0.45µm syringe filter. The viral supernatant was used immediately or snap-frozen and stored at -80°C until needed. For the production of retroviral particles, the same procedure was used, with the exception of using 10µg of the retroviral expression plasmid, 10µg of gag/pol (Addgene: #14887) and 2µg of pHCMV-EcoEnv (Addgene: #15802).

For transduction of target cells, viral supernatants were added to target cells and spinfected for 1.5-2h at 2000rpm and 37°C. After centrifugation viral supernatants were removed and cells were resuspended in regular cell culture medium. For retroviral transductions, plates were coated with RetroNectin (Takara Bio, Kusatsu, Japan) and viral supernatants were incubated with the cells overnight before replacement of medium.

**Mouse models of AML:** AML was induced using retroviral transductions with the MLL-AF9 oncogene as published previously (2, 3). In brief, lineage-, Kit+, Sca1+ cells from the bone marrow of donor mice were sorted using a FACS Aria II cell sorting system (BD Bioscience, Franklin Lakes, USA) and subsequently transduced with retroviral particles carrying MLL-AF9 (pMSCV-IRES-GFP vector system). After expansion for 4d, 20-50.000 cells were transplanted into sublethally irradiated recipient mice via tail vein injection. Transplanted mice were closely monitored, and engraftment was confirmed and monitored via analysis of the peripheral blood for GFP+ cells. Conditional knockout mice with floxed Ybx1 alleles were crossed to Mx1-Cre mice (B6.Cg-Tg(Mx1-cre)1Cgn/J, Jackson Laboratory, Bar Harbor, USA) and were injected with 3-5 doses poly(I:C) (GE Healthcare, Chicago, IL, USA) to induce gene deletion. Bone marrow (BM) of 6-12 weeks old Ybx1 F/F Mx1-Cre+, Ybx1+/+ Mx1-Cre+, Ybx1 F/F Cre- littermates was used. 1-2x$10^6$ whole bone marrow cells of diseased animals were used for secondary transplants. Sample sizes and experimental schedules were estimated based on previous experiences with leukemia models transformed by retroviral expression of MLL-AF9. Male and female mice were used for experiments in all groups, no specific method for randomization was used, investigators were not blinded. Variance was similar between the groups compared.

**Xenograft models of AML:** NOD.Cg-Prkdc$^{scid}$ Il2rg$^{tm1Wjl}$ Tg(CMV-IL3, CSF2, KITLG) 1Eav/MloySzJ (NSGS) were obtained from The Jackson Laboratory (Bar Harbor, ME, USA). 8-12-week-old adult mice were irradiated with 2Gy before transplantation. 2x$10^5$ MOLM-13 cells transduced with either of two YBX1 shRNAs or non-targeting control were injected intravenously via the tail vein. Female NOG mice NOD.Cg-*Prkdc*$^{scid}$ *Il2rg*$^{tm1Sug}$/JicTac were obtained from Taconic Bioscience (Rensselaer, NY, USA). 3x$10^4$ MOLM-13 cells harboring a CRISPR-Cas9 mediated knockout of YBX1 (YBX1-sgRNA1) or non-targeting control were transplanted into non-irradiated recipient animals. Engraftment of human cells (hCD45+) was analyzed by flow cytometry and mice were followed closely to monitor for disease onset. Sample sizes and experimental schedules were estimated based on previous experiences with leukemia induced by xeno-transplantation of MOLM13 cells. Male and female mice were used for experiments in all groups, no specific method for randomization was used, investigators were not blinded. Variance was similar between the groups compared.

**Label free proteome sample preparation**

Molm13 cells expressing sgRNA non-target control and sgRNA targeting Ybx1 were lysed in 1%SDC buffer (1%SDC, 100mM Tris pH8.5, 40mM CAA and 10mM TCEP), incubated on ice for 20 minutes, boiled at 95°C, sonicated for 15 mins on a Biorupter plus and heated again for 5minutes at 95°C (4) Proteins were digested with Trypsin (1:100 ratio) and LysC (1:100 ratio) for overnight at 37°C. To the peptides 5x volume Isopropanol/1% TFA was added and vortexed to stop the digestion. The peptides were de-salted on equilibrated styrenedivinylbenzene-reversed phase sulfonated (SDB-RPS) StageTips, washed once in isopropanol/1% TFA and twice with 0.2% TFA. Purified peptides were eluted with 60µl of elution buffer (80%, 1.25% NH4OH). The dried elutes were resuspended in MS loading buffer (3%ACN, 0.3% TFA) and stored at -20°C until MS measurement.

**Single shot LC-MS/MS measurement**

A nanoflow HPLC (EASY-nLC1000, Thermo Fisher Scientific) coupled online to an Orbitrap Exploris 480 Mass Spectrometer (Thermo Fischer Scientific) via a nans electrospray ion source was utilized for the sample analysis. Approximately 300 ng of peptides were loaded onto a 50-cm column with 75-µM diameter, packed in house with 1.9µM C18 ReproSil particles (Dr. Maisch GmbH). The column temperature was maintained using a homemade column oven at 60°C. The peptides were separated with the binary buffer system of 0.1% formic acid (buffer A) and 60% ACN plus 0.1%formic acid (buffer B), at a flow rate of 300nl/min. Peptides were eluted over a duration of 120 minutes with a gradient of 30% buffer B over 95 minutes and increased to 60% over for 5minutes. This was followed by a quick ramp up to 95% over 5 minutes and declined back to 5% over 5mins to re-equilibrate the column. Mass spectra was acquired in a data dependent mode. Briefly, with one full scan at a target of $3e^6$ ions (300-1650 m/z, R=60,000), followed by Top15 MS/MS scans with HCD (high energy collisional dissociation) (target 1e5 ions, maximum filling time 28ms, Isolation window 1.4 m/z, and normalized collision energy 27), detected in the Orbitrap at a resolution of 15,000. Dynamic exclusion 30s and charge exclusion (2-5) were enabled.

**MS data analysis and bioinformatics**

MS raw files were processed using Maxquant (5) version 1.5.5.2 supported by Andromeda search engine. The data was searched for proteins and peptides using a target-decoy approach with a reverse database against Uniprot Human (version 2016) fasta file with a false discovery rate of less than 1% at the levels of protein and peptide. Default settings were enabled such as oxidized methionine (M), acetylation (protein N-term), and carbamidomethyl (C) as fixed modification and Trypsin as enzyme specificity. A maximum of 2 missed cleavages was allowed and a minimum peptide length of seven amino acids set. The proteins were assigned to the same protein groups if two proteins could not be discriminated by unique peptides. The label-free quantification was performed using the MaxLFQ algorithm (6) and match between run feature was enabled for identification of peptide across runs based on mass accuracy and normalized retention times. For label free protein quantification minimum ratio count was set to 2. The Maxquant output table was analyzed in Perseus (7), prior to the analysis contaminants marked as reverse hits, contaminants and only identified by site-modification were filtered out.

**Fractionation of polysomes:**

MOLM13-Cas9 cells expressing either YBX1-sgRNA1 for knockout of YBX1, or a non-targeting control (sgLUC) were grown in T75 flasks in RPMI with 10% FBS and antibiotics. Cycloheximide (CHX) was added to a final concentration of 100µg/ml and incubated for 10min at 37°C. Subsequently, cells were collected, washed with DPBS containing same final concentration CHX (100µg/ml) and 500ul lysis buffer (10mM Tris-HCl, pH 7.4, 100mM KCl, 5mM MgCl2, 100ug/mL cycloheximide, 2mM DTT, 1% TritonX100) was added. After resuspension and 10 min incubation on ice cells were centrifuged for 10 min at 10000rpm and the supernatant was transferred to a clean tube and snap-frozen at -80°C. Sucrose gradients (10-50%) were prepared as previously described (8) and chilled at 4°C. Subsequently, 400µl was carefully loaded on top of the sucrose gradient. The lysates were spun in an ultracentrifuge for 80min. at 32 000 rpm (acceleration: slow, deceleration: off). After centrifugation fractions of 490µl were collected using a Piston Gradient Fractionator™ (Biocomp Instruments, Fredericton, Canada). Fractions were collected in 1.5ml Eppendorf tubes, snap frozen and stored at -80°C until used for RNA extraction and sequencing library preparation.

**Chromatin-Immunoprecipitation / ChIP-sequencing**

The cells were collected and washed twice in ice-cold PBS followed by crosslinking in 1% formaldehyde (methanol free) for 5 minutes. Excess of formaldehyde was quenched by addition of Tris pH 8.0 to 100nM final and Glycine 125 mM final. Cells were washed 3x in PBS and subsequently lysed in SDS-buffer (100mM NaCl, 50mM Tris-HCL pH 8.1, 5mM EDTA, 0.02% NaN3, 0.5% SDS) supplemented with Complete protease inhibitors (MilliporeSigma). Chromatin was spun at 15.000rpm in a tabletop centrifuge at room temperature, the supernatant was discarded and the translucent chromatin sediment was resuspended in IP-buffer (2 parts SDS-buffer + 1 part Triton dilution buffer (100mM NaCl, 100mM Tris-HCL pH 8.1, 5mM EDTA, 0.02% NaN3, 5% TritonX-100)) supplemented with protease inhibitors. Chromatin was stored on ice, transferred to 1ml Covaris sonication containers and chromatin from 20Mio cells was sheared using Covaris E220 for 20 min with power 140; duty 5; bursts 200/sec. After sonication the sheared chromatin was centrifuged at 15,000rpm at 4°C to remove debris. We then used 5ul of sheared chromatin to de-crosslink (>1h at 65°C) in order to test shearing efficacy (De-crosslinking buffer: 100mM NaHCO3, 200mM NaCl 1% SDS). Chromatin was considered passed QC if fragments in the range between 100-600bp were >90% of all fragments. For immunoprecipitation we used chromatin from 10x10^6 cells, 5ug of Anti-YBX1 antibody and 25ul of Protein-A magnetic beads (Dynabeads, Thermo Fischer Scientific). The antibody was conjugated to the beads for 1h on a rotator in 500ul of ChIP-dilution buffer (167mM NaCl, 16.7mM Tris-HCL pH 8.1, 1.2mM EDTA, 0.1% SDS, 1.1% TritonX-100) supplemented with 0.5% BSA, subsequently washed in ChIP dilution buffer and added to the sheared chromatin for rotation at 4°C overnight. After 16-18 hours the immune complexes were washed each 2 times for each 2 minutes in Mixed Micelle Buffer (150mM NaCl, 20mM Tris-HCL pH 8.1, 1mM EDTA, 0.02% NaN3, 1% TritonX-100, 0.2% SDS, 5.2% Sucrose), Buffer 500 (50mM Tris-HCL pH 8.1, 2mM EDTA, 0.02% NaN3, 1% TritonX-100, 0.5% deoxycholic acid) and LiCl-buffer (10mM Tris-HCL pH 8.1, 2mM EDTA, 0.02% NaN3, 25mM LiCl, 0.5% NP-40, 2.5% deoxycholic acid) detergent solution. After a final wash in 1x TE-buffer beads were resuspended in 100ul De-crosslinking buffer and incubated for 4-16h at 65°C before purification using AmpureXP beads (1:1.2 ratio). ChIP- or input- DNA was used for Illumina compatible library generation using ThruPlex DNA-seq kit (Takara) with 10 to 14 cycles of amplification and the use of single indexing barcodes. Sequencing was performed as 37bp paired end sequencing on a NextSeq500 platform (Illumina). Raw Illumina NextSeq BCL files converted to FASTQs using Illumina bcl2fastq v02.14.01.07 and reads trimmed using Trimmomatic v0.36 (phred quality threshold 33) and uploaded to the to the Basepair-server (basepairtech.com). Alignment and ChIP-seq QC was performed on the basepair platform (Bowtie2). Peak calling was performed using MACS (v.1.4) within the basepair platform utilizing the default parameters.

**RNA-Immunoprecipitation**

30 Mio cells were washed 3x in PBS and lysed in 500ul RIP-buffer (150mM KCL, 25mM Tris-HCL pH 7.5, 5mM EDTA, 0.5mM DTT, 0.5% NP40, Complete protease inhibitors, 100U/ml RNAsin RNase-inhibitor). Lysis was performed for 20min at 4°C on a rotator. Subsequently, debris was removed by spinning at 15,000prm for 10min. at 4°C. 5ug of Anti-YBX1 antibody per sample was conjugated to 25ul of Protein-A magnetic beads (Dynabeads, Thermo Fischer Scientific) for 1h on a rotator in 500ul of RIP-buffer supplemented with 0.5% BSA. The antibody-bead conjugate was added to the lysate and incubated on a rotator for 12-16h at 4°C. Subsequently, beads were washed 4 times in 1ml of RIP-wash-buffer (200mM KCL, 20mM Tris-HCL pH 7.5, 0.2mM EDTA, 1mM MgCl2, 20% Glycerol,

0.05% NP-40, 50U/ml RNAsin RNase-inhibitor). After the washes, beads were resuspended in 70ul of Proteinase-K-buffer (10mM Tris-HCL pH 8.2, 50mM NaCl, 5mM EDTA, 0.5% SDS, 2ug/ul Proteinase K, 200U/ml RNAsin) and incubated at 50°C for 30min. Subsequently, beads were removed using a magnet and liquid was added to 350ul RLT-buffer (RNeasy kit, Qiagen) and RNA extraction was performed according to the manufacturer's instructions.

**Histological imaging of mouse organs:**

The HE-stained paraffin-sections were imaged by employing an automated whole slide scanning microscope (Axio Scan.Z1, Carl Zeiss Microscopy GmbH) equipped with a 10x objective (Plan-Apochromat / 0.45) and a color camera (HV-F202SCL, Hitachi). Tiled images were processed and analyzed using the ZEN software (blue edition, version 2.3, Carl Zeiss Microscopy GmbH).

**Western Blotting:**

Cells were washed twice with PBS and 2 Mio cells were lysed in 200µl of Pierce™ IP Lysis Buffer (Thermo Fischer Scientific) for 10min. on ice. The lysate was spun down at 15.000rpm for 10min. in a tabletop centrifuge (cooled to 4°C) and supernatant was stored at -80°C or immediately used for gel electrophoresis. Per lane on a gel, 20µl lysate was mixed with 30µl Laemmli buffer freshly supplemented with DTT (10mM final concentration). The lysate was cooked at 95°C for 10min, before loading on 45µl per lane on a NuPAGE Bis-Tris 4-12% gradient gel (Thermo Fischer Scientific). Lysates were separated at a current of 100-120V in NuPAGE™ MOPS SDS Running Buffer (Thermo Fischer Sientific) and Transfer was performed using the iBLOT semidry transfer system (Thermo Fischer Scientific) for 9min. at 20V). The membranes were blocked in TBST + 5% BSA for 1h and stained with primary antibodies (1ug/ml) overnight at 4°C. On the next day, membranes were washed 3x in for 10min. in TBST and stained with HRP-labeled anti-Rabbit or anti-mouse secondary antibody (1:2000 dilution, Cell Signaling Technologies) in TBST + 5% dry milk. Membranes were washed 3x for 10min. in TBST and imaged using Immobilon Western Chemiluminescent HRP Substrate (MilliporeSigma) and images were taken on an Amersham Imager 600 (GE Healthcare).

**Primary patient samples**

All patient samples and healthy donor controls derived during routine biopsies (peripheral blood, bone marrow aspirates and biopsies) and investigated in this study were obtained after informed consent and according to the Helsinki declaration from the Tumor Banks in Jena and Magdeburg, Germany. Scientific protocol, patient information and patient approval forms have been approved by the respective local ethics committees ('Ethics Committee, University Hospital Jena' no. 4753/04-16 and 'Ethics Committee, Medical Faculty, OvGU Magdeburg' no. 115/08)

**List of antibodies used in this study:**

| Target | Isotype | Manufacturer / Cat# | clone |
|---|---|---|---|
| YBX1 | Rabbit IgG | Abcam / ab76149 | recombinant |
| c-Myc | Mouse IgG | Abcam / ab56 | 9E11 |
| H2A.Z | Rabbit IgG | Abcam / ab150402 | recombinant |
| GAPDH | Rabbit IgG | Cell Signaling Technologies / #2118 | 14C10 |
| β-Actin | Rabbit IgG | Cell Signaling Technologies / #4970 | 13E5 |
| Anti-rabbit HRP | Goat IgG | Cell Signaling Technologies / #7074 | polyclonal |
| Anti-mouse HRP | Horse IgG | Cell Signaling Technologies / #7076 | polyclonal |
| eIF4B | Rabbit IgG | Cell Signaling Technologies / #3592 | polyclonal |
| EEF2 | Rabbit IgG | Cell Signaling Technologies / #2332 | polyclonal |
| eIF3D | Rabbit IgG | Bethyl Laboratories / A301-759A | polyclonal |
| eIF3L | Rabbit IgG | Bethyl Laboratories / A304-754A | polyclonal |
| RPS15 | Rabbit IgG | Bethyl Laboratories / A305-042A | polyclonal |
| RPS15A | Rabbit IgG | Bethyl Laboratories / A304-990A | polyclonal |
| IDH3B | Rabbit IgG | Bethyl Laboratories / A305-500A | polyclonal |
| CD11b | Rat IgG – BV421 conjugate | Biolegend / 101235 | M1/70 |
| Gr-1 | Rat IgG – APC conjugate | Biolegend / 108412 | RB6-8C5 |
| Sca-1 | Rat IgG – PE conjugate | Biolegend / 108108 | D7 |
| cKit | Rat IgG – APC conjugate | Biolegend / 105812 | 2B8 |
| human CD45 | Mouse IgG – PE conjugate | Biolegend / 304039 | HI30 |
| BrdU | Mouse IgG – APC conjugate | BD Biosciences / 552598 | B44 |

**List of Taqman gene expression assays used in this study:**

| Target | Cat # | Dye | Manufacturer |
|---|---|---|---|
| Beta2 Microglobulin | Hs00984230 | FAM-MGB | Thermo Fischer Scientific |
| EIF4B | Hs04368067 | FAM-MGB | Thermo Fischer Scientific |
| EIF3L | Hs00275016 | FAM-MGB | Thermo Fischer Scientific |
| EIF3D | Hs01044815 | FAM-MGB | Thermo Fischer Scientific |
| EIF3D | Hs00157330 | FAM-MGB | Thermo Fischer Scientific |
| RPL4 | Hs03044646 | FAM-MGB | Thermo Fischer Scientific |
| RPL6 | Hs03044365 | FAM-MGB | Thermo Fischer Scientific |

**List of single-guide RNAs used in this study:**

| sgRNA | Species | sgRNA sequence | Vector system |
|---|---|---|---|
| Luciferase_sgRNA | Photinus pyralis | GATTCTAAAACGGATTACCA | pUSEPR (RFP) |
| RPA3_sgRNA1 | human | GGTTGGAAGAGTAACCGCCA | pUSEPR (RFP) |
| Rpa3_sgRNA1 | mouse | TGTTACCACAGTATATCGAC | pUSEPR (RFP) |
| YBX1-sgRNA1 | human | GTCTTGCAGGAATGACACCA | pUSEPR (RFP) + pLKO5.GFP |
| YBX1-sgRNA1 | human | GCAAATGTTACAGGTCCTGG | pUSEPR (RFP) |
| YBX1-sgRNA1 | human | ATAGACGCTATCCACGTCGT | pUSEPR (RFP) |
| Ybx1-sgRNA1 | mouse | AAATGGTTCAATGTAAGGAA | pUSEPR (RFP) |
| Ybx1-sgRNA2 | mouse | GTCTTACAGGAATGACACCA | pUSEPR (RFP) |
| Ybx2-sgRNA1 | mouse | GCTATGCCCCTAATCGACGT | pUSEPR (RFP) |
| Ybx2-sgRNA2 | mouse | CTAATCGACGTAGGTTCCGG | pUSEPR (RFP) |
| Ybx3_sgRNA1 | mouse | GGAATCCGACTTACCGCCCG | pUSEPR (RFP) |
| Ybx3_sgRNA2 | mouse | AGAGAACATACATTACGGGG | pUSEPR (RFP) |
| Carhsp1_sgRNA1 | mouse | CCCTACAGAAGTCTGATGCG | pUSEPR (RFP) |
| Carhsp1_sgRNA2 | mouse | GTCAGGACCACCATCAGCTG | pUSEPR (RFP) |
| Csdc2_sgRNA1 | mouse | CACGCCTGAGAATGGCTCCG | pUSEPR (RFP) |
| Csdc2_sgRNA2 | mouse | AGAGTCTACATTACCGCCAG | pUSEPR (RFP) |
| Csde1_sgRNA1 | mouse | TGACGAAGAACCATTAGACG | pUSEPR (RFP) |
| Csde1_sgRNA2 | mouse | TTGAAGTATCATCTGACCGG | pUSEPR (RFP) |
| Lin28a_sgRNA1 | mouse | GTGCACAAAGACGTCCACCG | pUSEPR (RFP) |
| Lin28a_sgRNA2 | mouse | TGCACAAAGACGTCCACCGG | pUSEPR (RFP) |
| Lin28b_sgRNA1 | mouse | GCACGTTGAACCATTTACAG | pUSEPR (RFP) |
| Lin28b_sgRNA2 | mouse | CATCTCCATGATAAGTCGAG | pUSEPR (RFP) |

## Supplementary figure legends:

**Supplementary Figure 1: A)** Schematic showing the experimental procedure underlying the flow-cytometry-based CRISPR-competition assay. **B)** Box plots showing relative protein expression of cold shock protein family members among cell lines from the cancer cell line encyclopedia (Mass-spec proteome analysis, data derived from depmap.org). Statistical analysis via unpaired t-test, * $p < 0.05$, ** $p < 0.01$, ***$p < 0.001$. **C)** Bar graphs showing the relative gene expression of cold shock protein family members in patients with different types of AML, compared to hematopoietic stem cells (HSC) and mononuclear cells (PMN). Statistical analysis via unpaired t-test, ** $p < 0.01$, ***$p < 0.001$.

**Supplementary Figure 2: A)** Bar graphs showing cell counts (top panels, counted by hematocytometer) and % of dead cells (bottom panels, Sytox-blue dead cell assay, measured by FACS) in a set of 8 different human AML cell lines after genetic inactivation of YBX1 using RNAi (shRNA1/2) on 4 consecutive days. **B)** Surface expression of the differentiation markers CD11b and CD11c on the same set of cell lines after genetic inactivation of YBX1 using RNAi (shRNA1/2).

**Supplementary Figure 3: A)** Bar graphs showing the number of AML colonies in a methylcellulose-based colony-formation assay in MLL-aF9 transformed murine LSK-derived AML cells after genetic inactivation of YBX1 by RNAi using 3 different shRNAs or non-targeting control. The numbers of weeks on the X-axis correspond to the rounds of re-plating to assess for self-renewal capacity. B) Survival curves of mice transplanted with MLL-AF9 driven AMLs that have a reduced expression of YBX1 mediated by RNAi (shYBX1) compared to YBX1 proficient leukemias (shNT). **C)** Bar graphs showing the peripheral blood chimerism of YBX1+/- (HET) cells compared to WT in a competitive repopulation assay in primary (left) and secondary (right) recipients each cohort followed over 16 weeks after transplantation. **D)** Relative abundance of hematopoietic stem- and progenitor cell populations in adult YBX1+/- (HET) mice compared to WT. **E)** Bar graphs showing the % of GFP+ cells in mice harboring a conditional YBX1-/- leukemia (blue bars) compared to YBX1 +/+ controls (grey bars) at 2, 4, 6 and 8 weeks post transplantation in primary recipient animals. **F)** Bar graphs showing the % of GFP+ cells in mice harboring a conditional YBX1-/- leukemia (blue bars) compared to YBX1 +/+ controls (grey bars) at 2, 4 and 6 weeks post transplantation in secondary recipient animals.

**Supplementary Figure 4: A)** Heatmap derived from hierarchical clustering of z-scores of all genes that were differentially expressed (all genes with an adjusted p-value < 0.05) at 7 days in MOLM13 cells harboring a CRISPR-Cas9-mediated YBX1-ko. **B)** Pie charts showing the distribution of YBX1 peaks in ChIP-sequencing among genes and intergenic regions (left) and within the gene-bound regions the distribution over promotors, exons and introns (right). **C)** Mapping of YBX1-ChIP-targets to differentially expressed genes at 7 days post YBX1-ko.

**Supplementary Figure 5: A)** Bar graphs visualizing the beta scores of RPL3, RPS8, RPS27, EIF3C, DDX51 and UTP15 in control MOLM13 cells compared to YBX1-ko cells. **B)** Pie charts showing overlap between RNA-sequencing results at day 7 vs. day 14 post lentiviral transduction with YBX1-targeting guide RNAs. **C)** Bar graphs showing the results from quantitative real-time PCR validating key targets that were found to be affected by YBX1-ko after 7d in RNA sequencing. Validation was performed in MOLM13 and OCI-AML2 cells. Statistics was performed using an

unpaired T-test. **D)** Western blotting analysis in MOLM13 cells to validate differential expression of translation initiation- and elongation factors that were identified as differentially expressed on the mRNA level in RNAseq and quantitative real-time PCR. Genetic inactivation of YBX1 was performed using 3 different single-guide RNAs. **E)** Western Blot showing forced expression of EIF4B in MOLM13 cells. EIF4B cDNA was cloned into the pLEX vector system and cells were selected with puromycin. F) Bar graphs showing the results of a cell competition assay in MOLM13 cells with forced expression of EIF4B or empty vector control after genetic inactivation of YBX1. The competitive disadvantage of YBX1 inactivation could not be rescued by EIF4B overexpression. **G)** Western Blot knockout control showing the protein expression of YBX1 in 4 replicate cell cultures that were subsequently used for lysis and polysomal fractionation. **H)** Western blotting analysis in MOLM13 cells to validate selected candidate genes, that were found to be depleted from polysomal chains upon YBX1-inactivation. Genetic inactivation of YBX1 was performed using 3 different single-guide RNAs. **I)** Volcano plot showing differentially expressed genes from RNAseq at 5 days after shRNA mediated knockdown of YBX1. All genes with an adjusted p-value below 0.05 and a fold change greater than 1.5 were considered differentially expressed.

**Supplementary Figure 6:** Absorbtion traces derived from the polysomal fractionation process. Cell lysates were fractionated using a sucrose gradient. After centrifugation fractions of 490µl were collected using a Piston Gradient Fractionator™ (Biocomp Instruments, Fredericton, Canada). The higher fractions highlighted in blue were pooled as "polysomal" fractions for RNAseq analysis.

**Supplementary Figure 1:**

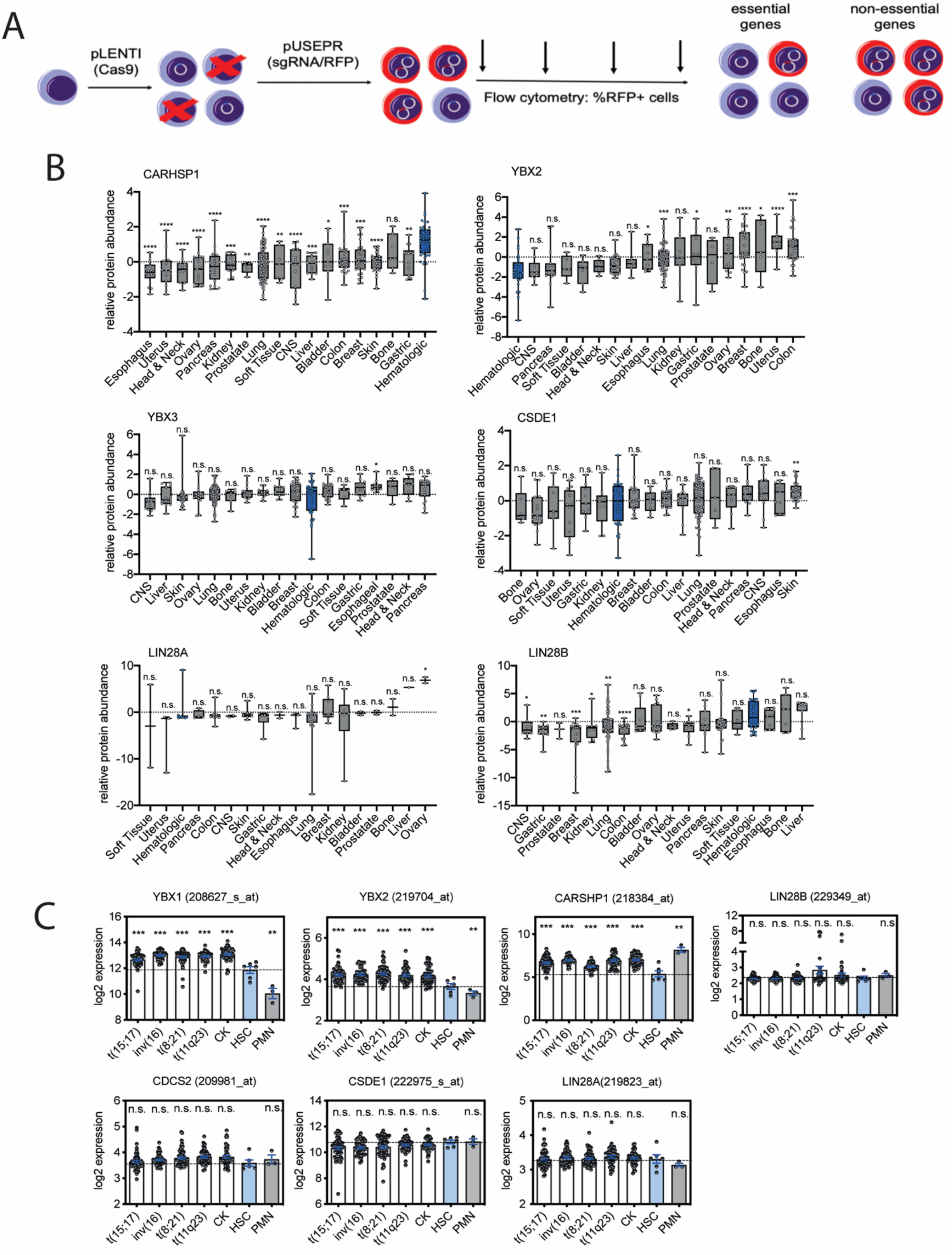

**Supplementary Figure 2:**

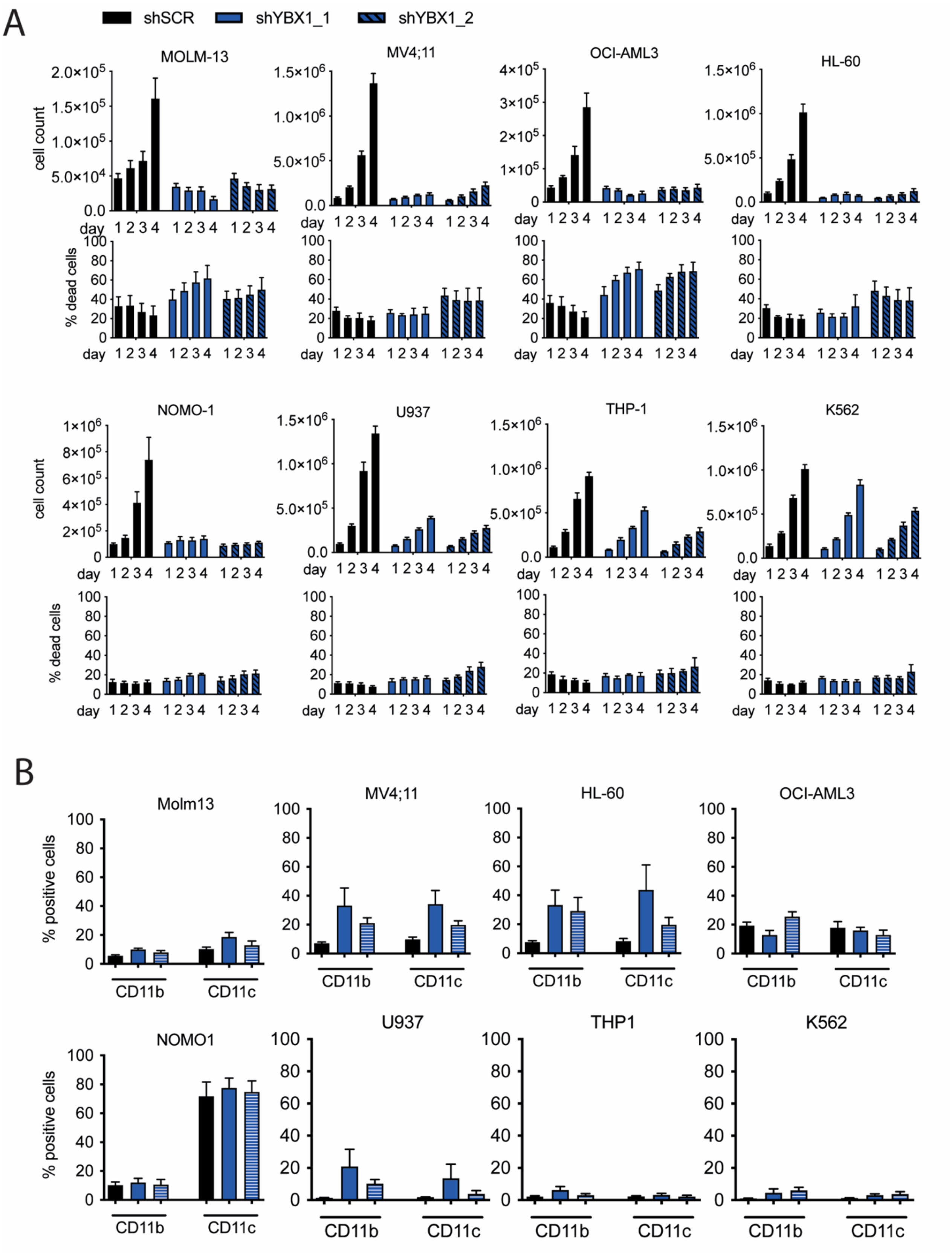

**Supplementary Figure 3:**

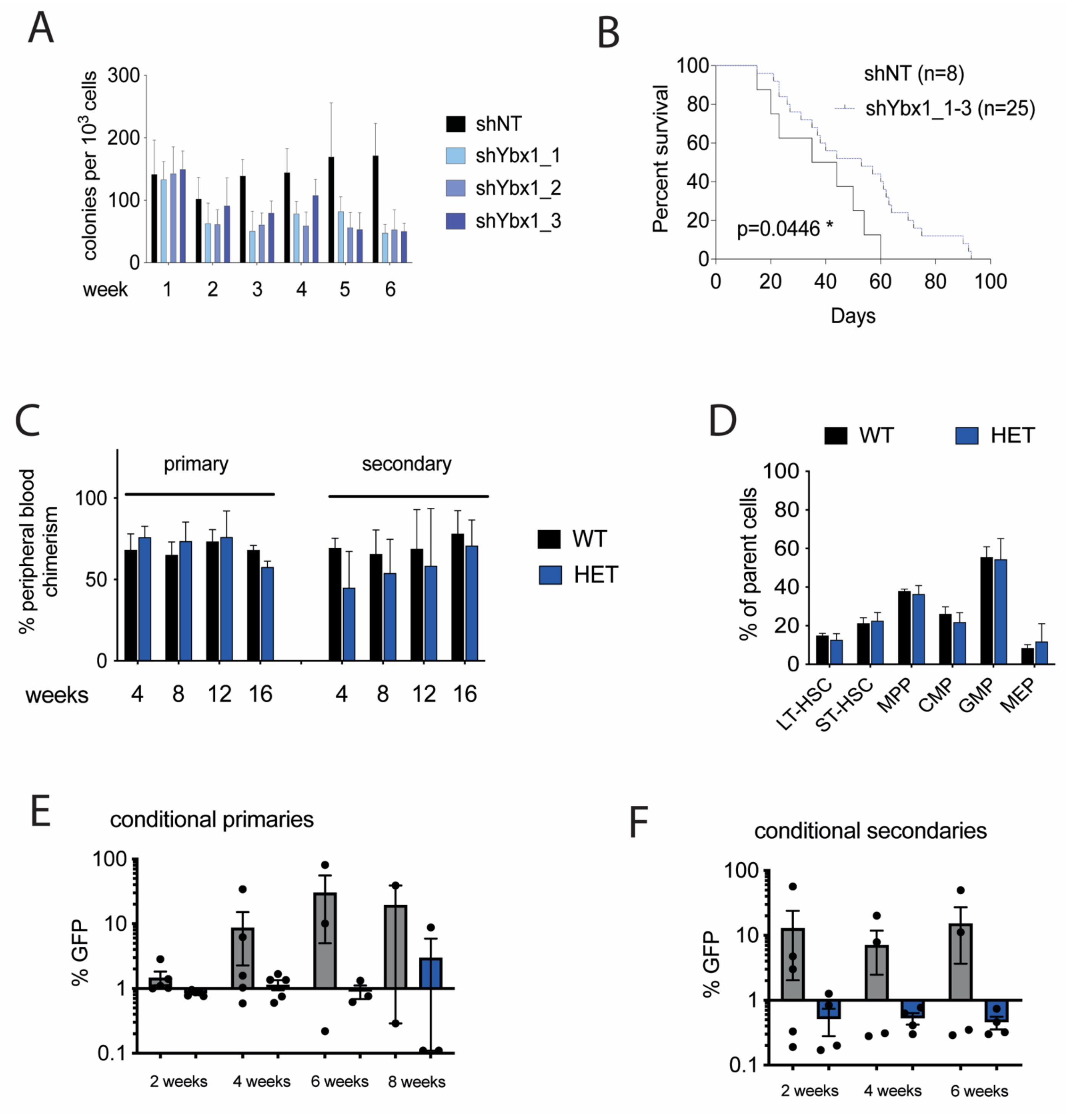

**Supplementary Figure 4:**

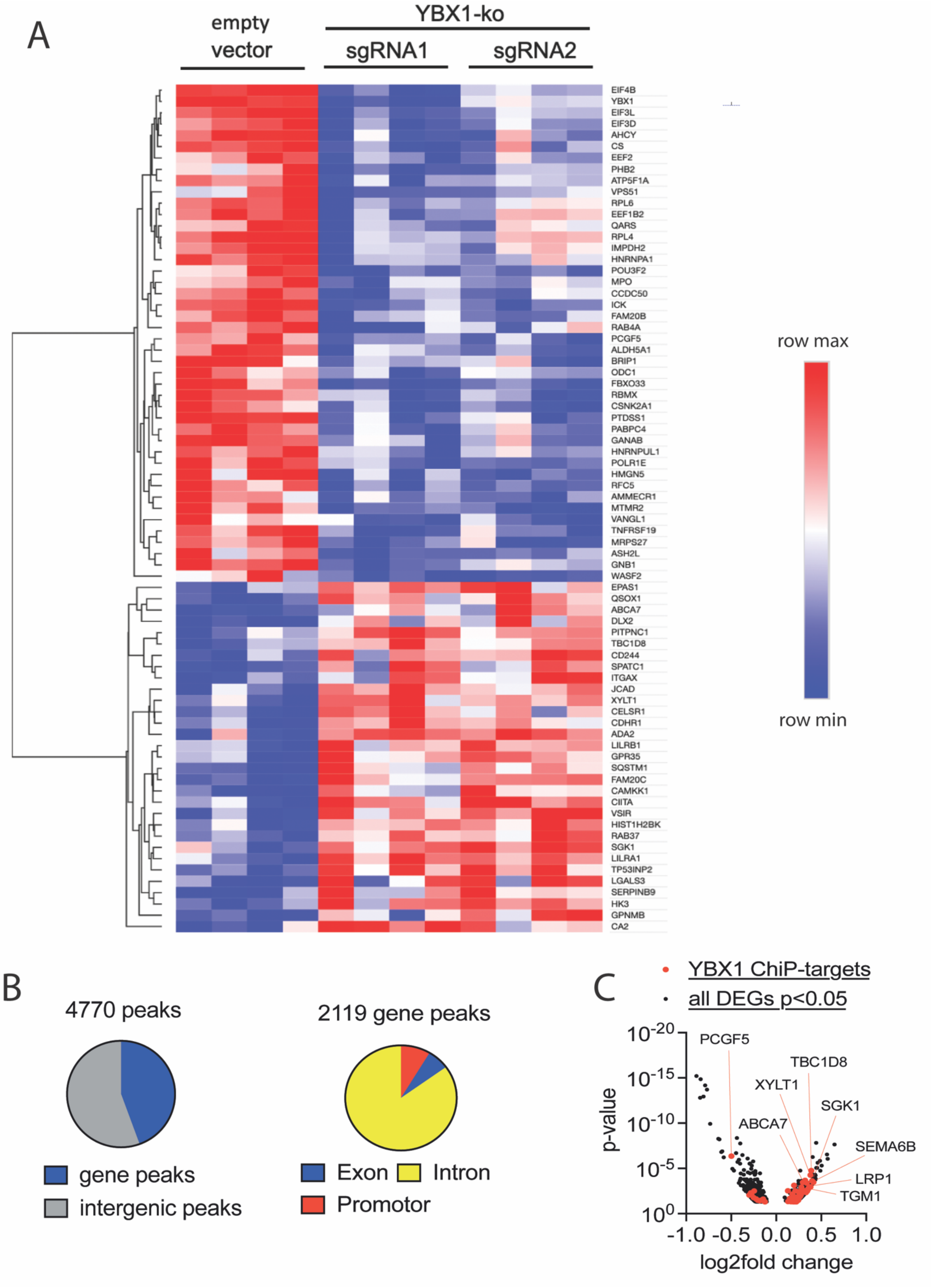

**Supplementary Figure 5:**

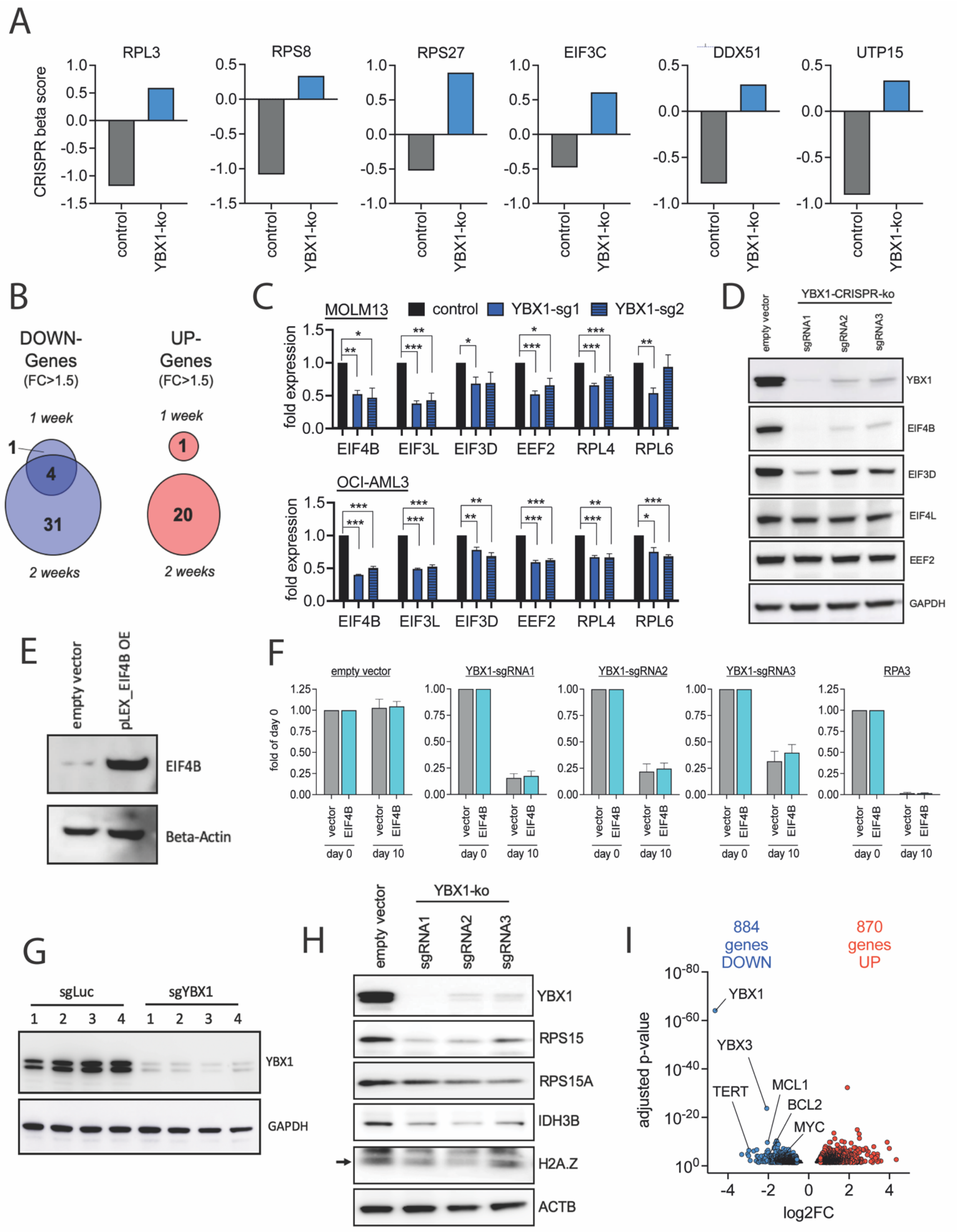

**Supplementary Figure 6:**

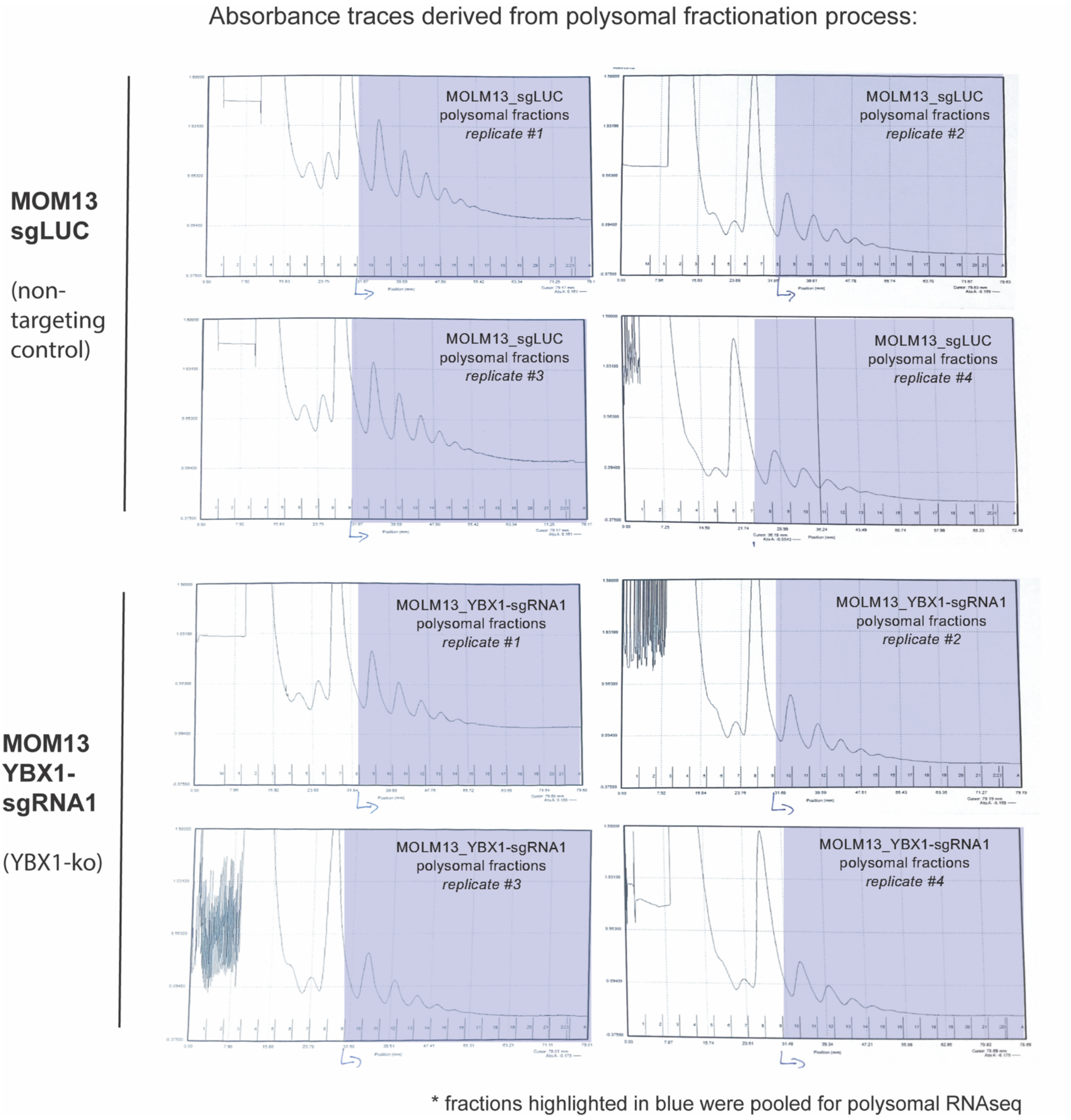

**References**

1. Uckelmann HJ, Kim SM, Antonissen NJC, Krivtsov AV, Hatton C, McGeehan GM, et al. MLL-Menin Inhibition Reverses Pre-Leukemic Progenitor Self-Renewal Induced By NPM1 Mutations and Prevents AML Development. Blood. 2018;132(Suppl 1):546-.

2. Heidel FH, Bullinger L, Feng Z, Wang Z, Neff TA, Stein L, et al. Genetic and pharmacologic inhibition of β-catenin targets imatinib-resistant leukemia stem cells in CML. Cell Stem Cell. 2012;10(4):412-24.

3. Mohr J, Dash BP, Schnoeder TM, Wolleschak D, Herzog C, Tubio Santamaria N, et al. The cell fate determinant Scribble is required for maintenance of hematopoietic stem cell function. Leukemia. 2018;32(5):1211-21.

4. Kulak NA, Pichler G, Paron I, Nagaraj N, Mann M. Minimal, encapsulated proteomic-sample processing applied to copy-number estimation in eukaryotic cells. Nat Methods. 2014;11(3):319-24.

5. Cox J, Mann M. MaxQuant enables high peptide identification rates, individualized p.p.b.-range mass accuracies and proteome-wide protein quantification. Nat Biotechnol. 2008;26(12):1367-72.

6. Cox J, Hein MY, Luber CA, Paron I, Nagaraj N, Mann M. Accurate proteome-wide label-free quantification by delayed normalization and maximal peptide ratio extraction, termed MaxLFQ. Mol Cell Proteomics. 2014;13(9):2513-26.

7. Tyanova S, Temu T, Sinitcyn P, Carlson A, Hein MY, Geiger T, et al. The Perseus computational platform for comprehensive analysis of (prote)omics data. Nat Methods. 2016;13(9):731-40.

8. Pringle ES, McCormick C, Cheng Z. Polysome Profiling Analysis of mRNA and Associated Proteins Engaged in Translation. Curr Protoc Mol Biol. 2019;125(1):e79.
